# Evolutionary genetics of the mitochondrial genome: insights from *Drosophila*

**DOI:** 10.1093/genetics/iyad036

**Published:** 2023-05-12

**Authors:** Damian K Dowling, Jonci N Wolff

**Affiliations:** School of Biological Sciences, Monash University, Melbourne, Victoria 3800, Australia; School of Biological Sciences, Monash University, Melbourne, Victoria 3800, Australia

**Keywords:** mitochondria, mtDNA, mitonuclear, mito-nuclear, cytonuclear, Mother's Curse, heteroplasmy, experimental evolution, FlyBook

## Abstract

Mitochondria are key to energy conversion in virtually all eukaryotes. Intriguingly, despite billions of years of evolution inside the eukaryote, mitochondria have retained their own small set of genes involved in the regulation of oxidative phosphorylation (OXPHOS) and protein translation. Although there was a long-standing assumption that the genetic variation found within the mitochondria would be selectively neutral, research over the past 3 decades has challenged this assumption. This research has provided novel insight into the genetic and evolutionary forces that shape mitochondrial evolution and broader implications for evolutionary ecological processes. Many of the seminal studies in this field, from the inception of the research field to current studies, have been conducted using *Drosophila* flies, thus establishing the species as a model system for studies in mitochondrial evolutionary biology. In this review, we comprehensively review these studies, from those focusing on genetic processes shaping evolution within the mitochondrial genome, to those examining the evolutionary implications of interactions between genes spanning mitochondrial and nuclear genomes, and to those investigating the dynamics of mitochondrial heteroplasmy. We synthesize the contribution of these studies to shaping our understanding of the evolutionary and ecological implications of mitochondrial genetic variation.

## Introduction

Since their formal discovery in the 1890s, mitochondria were for the most part studied by biochemists and physiologists seeking to understand the biochemical and metabolic pathways regulating mitochondrial respiration (Altmann 1890; Green 1959; Schneider 1959; Ernster and Schatz 1981). During this time, mitochondria were viewed as unusual cytological bodies because, unlike any other animal cell componentry, they exhibited an outer double membrane (Sjostrand 1953). A revolution in the study of mitochondria occurred in the early 1960s with the discovery of mitochondrial DNA (mtDNA) (Nass and Nass 1963; Luck and Reich 1964; Schatz *et al.* 1964), a finding that led to the realization that mitochondria were organelles of endosymbiotic origin (Margulis 1970). This finding elevated mitochondria from being viewed as peculiar cellular components, to organelles that may hold insights into the evolution of eukaryogenesis, setting the stage for geneticists to commence investigations into the functionality and phylogenetic origins of the mitochondrial genome over subsequent decades. Following the increasing attention from the scientific community and the development of the molecular toolkit required to decipher and manipulate mtDNA, the field of mitochondrial genetics emerged (Linnane *et al.* 1971; Ernster and Schatz 1981).

The fruit fly, *Drosophila melanogaster*, was first proposed as a genetic model by Charles W. Woodworth at the start of the 20th century and has since become one of the most widely used and genetically best described eukaryotic organisms (Ashburner *et al.* 2005). Here, we review how insights from *Drosophila* research were pivotal to establishing and advancing the field of mitochondrial evolutionary genetics. We focus on overviewing research into genes and genetic variation within the mtDNA itself, covering studies that have broadly tested a basic assumption and null hypothesis in the field—that the genetic variation that has accumulated and that segregates within the mitochondrial genome has evolved under expectations of neutral theory.

Our goal is to provide an account of the history of the contribution of *Drosophila* to the field of mitochondrial evolutionary genetics and to illustrate how this research has helped shape the development of the field over the past 50 years. Research during this time has catalyzed a marked shift in the way we perceive and study the mitochondrial genome. Once viewed as a peripheral player in evolutionary and biomedical research, an accumulating base of evidence has shown that the genetic variation that segregates within the mitochondrial genome can be associated with manifold effects on components of organismal health and life history and on the penetrance of metabolic diseases. We note that much of the primary research we discuss in this review was published in *Genetics*, emphasizing not only the role of the genetic model *Drosophila* but also the role of the *Genetics Society of America*, in establishing the field of mitochondrial evolutionary genetics.

### Early beginnings

The years following the discovery of mtDNA saw concerted efforts by biologists to describe the heredity, function, gene content, and transcriptional and translational properties of mtDNA (Gibor and Granick 1964; Nass *et al.* 1965; Granick and Gibor 1967; Roodyn and Wilkie 1968; Borst and Kroon 1969; Nass 1969). It quickly became clear that animal mitochondrial genomes (at least those of bilaterian metazoans) were generally highly conserved, revealing distinctive similarities in size and base content across taxa, with mitochondrial genes forming an essential component of the genetic repertoire of eukaryote cells (Nass 1969; Sager 1972; Wallace 1982). Although gene arrangement was found to differ between taxa, the vast majority of mitochondrial genomes was found to host an identical set of genes: 22 transfer RNAs (tRNAs) and 2 ribosomal subunits (rRNAs) of the mitochondrion's own translational machinery and 13 protein-coding genes providing essential components of the ATP-generating electron transport system embedded in the mitochondrial inner membrane (Wolstenholme and Clary 1985).

The first published mitochondrial genome (mitogenome) was that of humans, a feat that required substantial efforts because technology at the time was reliant on the cloning of restriction fragments into appropriate vectors prior to sequencing and deciphering of fragments using large polyacrylamide gels (Sanger *et al.* 1977; Sanger and Coulson 1978; Anderson *et al.* 1981). Mitogenome sequences of *Drosophila* species soon followed this, reflecting the importance of *Drosophila* as an emerging genetic model. The mitogenome of *Drosophila yakuba* was the first fully sequenced mitogenome of an invertebrate species (Clary *et al.* 1982, 1983; Clary and Wolstenholme 1983a, 1983b, 1985). Sequencing of the mitochondrial coding region of *D. melanogaster* occurred shortly after (de Bruijn 1983; Garesse 1988), but the full sequence remained unknown until 1995 (Lewis *et al.* 1995). The long time lag separating the publication of the mitogenomes of these 2 *Drosophila* species is likely explained by the significant length differences of the AT-rich regions of each species— measuring ∼1 kb for *D. yakuba* but 5 kb for *D. melanogaster*, rendering sequencing of the latter species more difficult. Technological advances, such as polymerase chain reaction (PCR) in the late 1980s, accelerated sequencing efforts in ensuing years, but it was not until the inception of next-generation technologies that data acquisition was truly revolutionized (Saiki *et al.* 1988; Metzker 2010). At the time we embarked on writing this review, there were over 50,000 whole mitogenome sequences published in GenBank, many of which are of *Drosophilid* origin. Many more mitogenomes are available if one was to mine publicly available next-generation sequencing databases, which often contain mitogenomes as sequencing by-products in studies of nuclear gene expression (Smith 2013).

### The rise of mtDNA as a molecular marker

Soon after its discovery, researchers realized the prospective utility of mtDNA as a molecular marker for studies spanning the fields of phylogenetics, phylogeography, and systematics. This DNA became a gold standard marker of molecular diversity in animals, on account of several characteristics of mitochondrial genome inheritance that made the interpretation of polymorphism data simpler than that of polymorphisms of nuclear origin (Avise and Ellis 1986; Avise *et al.* 1987; Moritz *et al.* 1987; Harrison 1989). High copy number, small size, and ease of isolation made it technically feasible to extract high-quality DNA from even minute samples, and the apparent lack of recombination and paternal inheritance of metazoan mtDNA enabled researchers to track whole genealogies through evolutionary time with ease (Birky 1978).

Indeed, one of the key aspects of metazoan mitochondrial genomes that facilitated their utility in genealogical inference is their elevated mutation rates in comparison with most nuclear genes (Brown *et al.* 1979). The genome-wide mutation rate is generally 5 to 10 times higher for mitochondrial than nuclear genes (Brown *et al.* 1979), a value corroborated in *D. melanogaster* (Haag-Liautard *et al.* 2008). The high mutation rate and the lack of recombination of mitochondrial genes proved particularly useful with the inception of the *molecular clock* concept in the 1960s (Zuckerhandl and Pauling 1962; Wilson *et al.* 1977), which assumes a relatively constant rate of change for DNA and protein sequences within and across species, enabling the exploration of evolutionary time between divergence of taxonomic lineages within a phylogeny. Molecular dating has since become one of the most powerful and widely used tools in biology (Avise *et al.* 1987; Moritz *et al.* 1987; Kumar 2005). It assumes that the mutations that reach fixation within species (substitutions) and drive divergence between species generally follow a neutral or nearly neutral model of sequence evolution (Kimura 1968; Ohta 1973).

## The neutrality assumption

The neutral theory of molecular evolution predicts that most of the genetic variation found within and across species will be selectively neutral (not modifying phenotypic expression), shaped largely by drift and reaching fixation by chance (Kimura 1968, 1983, 1991; King and Jukes 1969). Such a model is pertinent when applied to the mitochondrial genome, given the products of the mtDNA sequence are critical to the regulation of energy conversion, and will therefore be expected to evolve under strong purifying selection. As such, it is envisaged that mutations arising in the mtDNA sequence that alter the biochemical phenotype would be deleterious in effect and quickly purged, leaving those that remain to segregate neutral or near neutral to selection.

In the sections that follow in this review, we will overview evidence from subdisciplines within the field of mitochondrial evolutionary biology, which together have tested the neutrality assumption for mtDNA variation, and that suggest capacity for a complex interplay of positive (Darwinian), balancing and negative (purifying) selection in shaping the accumulation and maintenance of the genetic variation within the mitochondrial genomes of *Drosophila*.

## Testing for signatures of nonneutrality in mitochondrial sequence data

A prediction to arise from neutrality theory is that the ratio of nonsynonymous substitutions (those causing changes in the amino acid sequence and hence functional) to synonymous substitutions (those that do not change the amino acid sequence and hence traditionally regarded as silent) found between species should equal the ratio of nonsynonymous to synonymous polymorphisms found within species (McDonald and Kreitman 1991). A neutrality index can be derived from this prediction, which measures the direction and degree to which amino acid variation within species departs from expectations of a neutral model (Rand and Kann 1996; Stoletzki and Eyre-Walker 2011). Ratios >1 indicate an excess of amino acid polymorphisms segregating within species relative to that expected under a strictly neutral model and indicate a strong influence of purifying selection in shaping the divergence in intraspecies and interspecies ratios, whereas ratios <1 indicate an excess in interspecies divergence of amino acid changing substitutions and strong influence of positive selection.

Studies to have calculated the neutrality index for mitochondrial genes in *Drosophila* have generally found that the strict neutrality prediction does not hold true for most protein-coding genes. Rather, the pattern for most mitochondrial genes is indicative of a general excess of polymorphisms within species, which ultimately do not reach fixation (Ballard and Kreitman 1994; Rand *et al.* 1994; Rand and Kann 1996; Cooper *et al.* 2015). These patterns are similarly reflected in analyses on humans and murids and across other animal taxa (Nachman *et al.* 1994, 1996; Nachman 1998). The findings are consistent with predictions of a model of nearly neutral evolution, in which the polymorphisms involved are assumed to confer slightly deleterious effects that are able to accumulate within species (Rand *et al.* 1994; Rand and Kann 1996; Cooper *et al.* 2015). Notwithstanding, such a pattern may also theoretically arise if mitochondrial polymorphisms were maintained under some form balancing selection, for example if epistatic interactions between polymorphisms in mitochondrial and nuclear genomes were under strong selection (mitonuclear fitness interactions) or if the polymorphisms were maintained under negative frequency-dependent selection. We address the evidence for balancing selection later in this review.

Further comparative studies of *Drosophila* mitogenomes revealed variation in evolutionary rates across different tRNA genes and protein-coding genes and across lineages (Ballard 2000a, 2000b; Drosophila 12 Genomes Consortium 2007; Montooth *et al.* 2009), with NADH dehydrogenase subunits accruing significantly more amino acid substitutions than those assembling the cytochrome c complex, as well as large interspecies variation in the number of nonsynonymous substitutions accrued across the phylogeny (Drosophila 12 Genomes Consortium 2007; Montooth *et al.* 2009). Differences were also reported in codon usage bias at synonymous sites across different mtDNA genes and haplotypes and differences in A/T representation in regions between coding genes (Ballard and Kreitman 1994; Ballard 2000b; Montooth *et al.* 2009), suggestive of weak selection on these sites. In sum, these studies testing for signatures of molecular selection on the *Drosophila* mitogenome revealed scope for weak and positive selection on various regions, presenting insights into the evolutionary processes shaping mitogenomes and raising some questions as to the reliability of the mitogenome as molecular marker for evolutionary inference (Ballard and Whitlock 2004; Galtier *et al.* 2009).

## Early evidence for nonneutrality and the capacity for cytonuclear fitness interactions to maintain polymorphism within populations

**Fig. 1.**
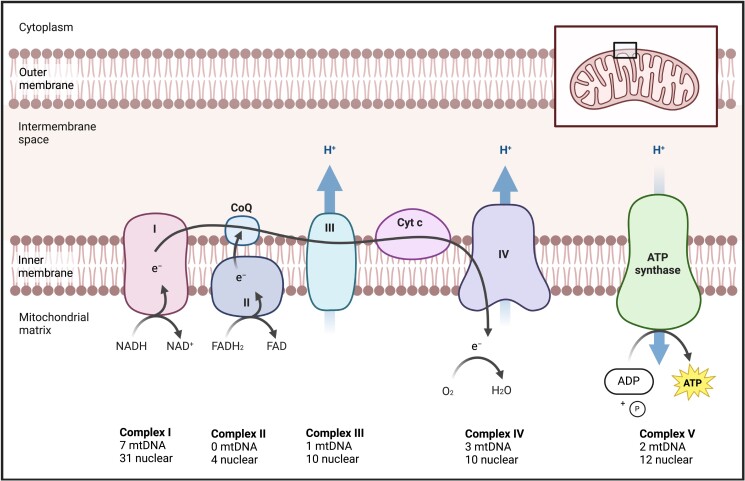
Schematic representation of the mETS, which generates ATP via OXPHOS. The system consists of 5 multisubunit enzyme complexes, each embedded within the mitochondrial inner membrane; complexes I and III–V are assembled by subunits encoded by mtDNA and nuclear DNA, while the subunits of complex II are solely encoded by nuclear DNA. Electrons (e^−^) extracted from food-derived substrates enter the system through electron carriers (NADH and FADH_2_) at complexes I and II, respectively, and flow through complex III via coenzyme Q (CoQ), then complex 4 via cytochrome c (Cyt c), fueling the pumping of protons (H^+^) from the mitochondrial matrix into the intermembrane space and creating a proton motive force (mitochondrial membrane potential). At complex IV, molecular oxygen acts as a terminal electron acceptor and is reduced to water. The mitochondrial membrane potential is dissipated at complex V through reentry of protons back into the mitochondrial matrix, driving the phosphorylation of ADP to ATP. Adapted from “Electron Transport Chain” by BioRender.com (2022). Retrieved from https://app.biorender.com/biorender-templates.

The mitochondrial electron transport system (mETS), which generates ATP via oxidative phosphorylation (OXPHOS), depends on coordination between genes distributed over both nuclear and mitochondrial genomes (Fig. 1). By the beginning of the 1980s, it was known that assembly of most of the protein complexes comprising the mETS was reliant on gene products derived from both genomes (Beale and Knowles 1979). Considering the central role of OXPHOS to cellular function in eukaryotes, it thus became clear that the mitochondrial genes contributing to OXPHOS are visible to selection and that there was capacity for the outcomes of selection to be mediated via epistatic interactions between these mitochondrial genes and those within the nuclear genome (Avise *et al.* 1987).

Indeed, it had long been known from observations of non-Mendelian cytoplasmic sterility that cytonuclear interactions could confer changes in phenotypic fitness in plants (Rhoades 1933; Edwardson 1956, 1970) and animals (including *Drosophila*; Caspari and Watson 1959; Ehrman 1963), but the underpinning mechanisms and knowledge of the inherited “cytoplasmic factor” remained largely unknown. Observations in fungi—of the *petite* and *poky* mutants of yeast and *Neurospora*, respectively—were among the first studies to link phenotypic changes directly to the mitochondria and later to the effects of mtDNA mutations themselves (Mitchell and Mitchell 1952; Ephrussi 1953; Silagi 1965; Lambowitz *et al.* 1976). Similar fitness effects associated with polymorphisms in mitochondrial genes were reported in early work on mouse cell lines harboring 2 distinct mitochondrial haplotypes in heteroplasmy (Slott *et al.* 1983). A cell line carrying a mutant haplotype that differed to the wild type by only 1 amino acid change in the ATP6 gene showed increased drug resistance to oligomycin, similar to findings observed earlier in yeast (Parker *et al.* 1968; Stuart 1970). One of the most striking findings of this study was that mutant haplotype frequencies in replicated subcultures of this oligomycin-resistant cell line increased over the course of the experiment from levels of ∼20% and stabilized at levels of 50%, suggesting balancing selection acting on the 2 haplotypes (Slott *et al.* 1983).

These early findings of nonneutrality of mtDNA in plants and animals, and maintenance of functional polymorphisms within cultures of cell lines, inspired evolutionary theoreticians in the 1980s to address the question of whether viability or fertility differences associated with varying combinations of cytoplasmic and nuclear genotype may facilitate the maintenance of nonneutral polymorphism. These theoreticians used 2 locus models (a cytoplasmic locus with 2 alleles and a nuclear locus with 2 alleles) to explore possible conditions under which cytonuclear fitness interactions could maintain joint polymorphisms at the interacting cytoplasmic (incorporating mtDNA, plastid, or other cytoplasmic genetic elements) and nuclear loci. The results of these models suggested only very limited capacity for the maintenance of such polymorphisms, primarily restricted to cases of strong differential selection on these loci within (across life stages) and between the sexes (Clark 1984; Gregorius and Ross 1984; Ross and Gregorius 1985; Babcock and Asmussen 1996, 1998). A later model by Rand *et al.* (2001) highlighted further conditions facilitating the maintenance of joint cytonuclear polymorphism—when the interacting nuclear loci were X chromosome linked, particularly when combined with increasing levels of paternal inheritance of the cytoplasmic gene (Rand *et al.* 2001). Implicit in many of these models was that when applied to metazoans, the interactive cytoplasmic locus would be mitochondrial in origin—thus, the models plausibly modeled conditions under which *mitonuclear* interactions would uphold stable polymorphism in each gene (Clark 1984; Babcock and Asmussen 1996, 1998; Rand *et al.* 2001).

This theoretical work inspired empirical tests to determine whether mitochondrial and nuclear variants, drawn from within panmictic populations, interacted to affect fitness components. All of the tests of this population genetic theory came from studies leveraging *D. melanogaster* (Clark and Lyckegaard 1988; Rand *et al.* 2001; Dowling *et al.* 2007; Friberg and Dowling 2008), owing to the experimental tractability of the model system and, in particular, the ease with which alternative cytoplasms could be placed alongside controlled and varying nuclear genetic backgrounds. Experimental generation of cytonuclear genotypes could be achieved either with the use of balancer chromosomes (containing inversions and rearrangements that prevent recombination between chromosomes) to substitute whole chromosomes between strains to engineer target cytonuclear genotypes (Roote and Prokop 2013) or through standard approaches of population introgression in which maternally inherited cytoplasms from 1 strain are introgressed into target nuclear backgrounds over sequential generations of backcrossing (Dowling *et al.* 2008).

The results of the early empirical studies generally aligned with predictions from the population genetic theory. Of those studies that specifically probed for interactions between cytoplasmic and nuclear genotypes sourced from within the same populations, Clark and Lyckegaard (1988) failed to detect evidence for within population cytonuclear interactions affecting 1 component of juvenile viability. The measure used was a chromosome segregation assay, in which individual larvae/pupae of target cytonuclear genotypes compete within vials with siblings carrying a visibly marked X chromosome, and proportions of wild type to marked X chromosome in each sex are then scored to provide a measure of relative juvenile fitness. The assay thus allows one to assess whether particular combinations of cytonuclear genotype affect the relative competitiveness of a wild-type X chromosome competing against a marked X-chromosome balancer. Subsequently, Rand *et al*. (2001) reported cytonuclear interactions for this same trait when specifically examining interacting nuclear variation confined to the X chromosome. Later, Dowling *et al*. (2007) and Friberg and Dowling (2008) focused on components of adult fitness—female fecundity and male competitive fertility, respectively. They reported cytonuclear interactions (sampling 25 cytoplasmic strains tested against 3 nuclear backgrounds) for female adult fitness (although these interactions were further contingent on interactions with the sampling block of the experiment, indicating a high level of context dependency in outcomes of the interactions), but not adult male fitness (Dowling *et al.* 2007; Friberg and Dowling 2008). Using the same panel of 25 cytoplasmic strains as used by Dowling *et al*. (2007) and Friberg and Dowling (2008), further research reported cytoplasmic genetic variation for life span (Maklakov *et al.* 2006), and follow-up enquiry indicated that at least 2 different mtDNA haplotypes segregated in the sampled population and were associated with life span differences (Dowling *et al.* 2009), albeit these studies were not designed to test for cytonuclear interactions on life span. Notably, studies of Rand *et al*. (2001) and those of Dowling *et al*. (2007) and Friberg and Dowling (2008) suggested outcomes of intrapopulation cytonuclear fitness interactions may be sex specific, in alignment with theoretical expectation that such interactions are more likely to promote stable joint polymorphism when selection differs across males and females.

Notwithstanding, these early empirical tests had relatively low inferential power to partition mitochondrial genotypic effects from other potential cytoplasmic sources of variance, making it difficult to extend their results to directly address the question of whether the complex intergenomic interactions reported in these studies reflected interactions between mitochondrial and nuclear genome. One of the reasons for this lower power is that generally, by necessity, studies screening for intrapopulation effects sampled random females from within the 1 lab population of *Drosophila* to establish their cytoplasmic strains, and it is unlikely that this approach would have captured more than just a few distinct mitochondrial haplotypes (Dowling *et al.* 2009). Even though some of the studies had eliminated the possibility of intracellular and therefore cytoplasmic-transmitted bacteria, such as *Wolbachia*, from confounding the results, either through antibiotic treatment of the strains used (Dowling *et al.* 2007; Friberg and Dowling 2008) or by verifying the absence of *Wolbachia* infection in sampled populations (Rand *et al.* 2001), the primary cytoplasmic genetic element driving the cytonuclear interactions detected in these studies remains open to question and could for instance include contributing variation of factors such as viruses and maternally loaded mRNAs (Clark 1985). For example, Rand *et al*. (2001) probed for cytoplasm by X-chromosome interactions within 3 distinct global populations of *Drosophila*, finding evidence in some but not all populations. Yet, once a population known to carry *Wolbachia* was removed from analysis, the occurrence of these interactions was limited to just males in 1 of the 2 other populations.

A second reason for the low inferential power of these studies is that, similar to many chromosome substitution studies conducted in *Drosophila* to date, the target genetic strains created have generally lacked independent biological replication at the level required to statistically partition genotypic from other confounding sources of variation—the level of the cytonuclear/mitonuclear genotype. That is, in numerous studies, each cytonuclear genotype was generally created just once (not created in replicate) and kept as a population. As such, any phenotypic differences detected between strains could be shaped by differences in cytonuclear genotype or by differences in any other sources of variance that might typically shape interindividual phenotypic variation across populations—by either environmental (e.g. shared environments of flies of each strain) or residual nuclear variation that may build up across the strains that share the same target nuclear genotype. This replication (or lack of genotype replication) issue continues to be relevant to numerous studies covered in this review and among contemporary studies of mitonuclear interactions; it represents an important methodological design consideration for future research in this field. Furthermore, a lack of replication at the level of the focal genotype (mito × nuclear interaction) may not only impede capacity to partition dedicated epistatic effects of mitochondrial and nuclear genetic variation from other sources of confounding variance but may also lead to nonoptimal specification of the error variances in the associated statistical models. In the absence of this replication, then instead of the error variance (reflected by the denominator degrees of freedom) representing the total number of mitonuclear strains used in an experiment, it may be represented either as the number of “shared environments” in which focal individuals are maintained prior to or during the experiments (e.g. “vial identity” is often specified as a random intercept in the associated statistical models) or as the total number of focal individuals assayed (in which case no random intercept is specified at all). Error variances at these levels may lead to pseudo-replication, inflation of test statistics, anticonservative *P*-values, and higher rates of Type 1 error than intended (Arnqvist 2020).

Thus, while the outcomes of intrapopulation studies of cytonuclear interaction are moderately consistent with the theoretical expectation that joint polymorphism is only expected under a limited set of specific conditions, inferences from these studies are somewhat inconclusive when applied specifically to the question of mitonuclear interactions, due to the methodological considerations discussed above. Encouragingly, studies that redress these limitations are beginning to emerge, for example through research into mitonuclear interactions in *Drosophila subobscura*. The *D. subobscura* model system provides several advantages in the study of intrapopulation mitonuclear dynamics: sympatric populations have been identified that harbor distinct mtDNA haplotypes; haplotype variation in this species exhibits higher levels of nucleotide divergence than found in the more often studied *D. melanogaster* (∼1.5% between the major haplotypes I and II in *D. subobscura* relative to 0.3% between haplotypes in *D. melanogaster*) (Latorre *et al.* 1986; Morrow *et al.* 2015), which presumably increases the likelihood of the haplotypes conferring differences in effect on phenotype; and signatures of linkage disequilibrium between certain nuclear regions harboring inversions and mtDNA haplotype have been reported in the species, suggestive of selection on mitochondrial and nuclear genotypic pairings (Oliver *et al.* 2002).

Accordingly, Kurbalija Novičić *et al*. (2015) created a panel of strains of *D. subobscura*, generated from isofemales lines collected from a single population, in which 3 distinct and well-known mtDNA haplotypes (haplotype I, II, and D; haplotypes I and II diverge at numerous sites in the nucleotide sequence, while D shares a similar allozyme sequence to haplotype I but is longer in length) were placed alongside 3 nuclear backgrounds, with each mitonuclear combination created in 10 replicates. They then measured flies of each strain for mass-specific metabolic rate, as gauged by CO_2_ production, across 3 cohorts of flies of young adult age, for each sex and replicate. While they failed to detect effects of nuclear genetic background, or mitonuclear interactions, on metabolic rate, they did detect effects of mtDNA haplotype (with differences in metabolic rate of up to 20% across the 3 haplotypes), which were partially contingent on the sex of the flies. Specifically, flies harboring the D haplotype exhibited lower metabolic rate than those bearing I or II haplotypes. Flies carrying the I haplotype differed to those with the II haplotype, but this effect was contingent on sex—males with haplotype I exhibited reduced metabolic rate than those with haplotype II, but the pattern was reversed in females, with those with haplotype I exhibiting increased metabolic rate (Kurbalija Novičić *et al.* 2015). Sex differences in mitochondrial genotypic effects on fitness have been increasingly observed and are overviewed in the *Sex specificity of mitochondrial haplotype effects* section of this review.

A follow-up study by Jelic *et al*. (2015) on these same strains reported mitonuclear interactions affecting offspring sex ratios, as well as sex-specific outcomes of mitonuclear interactions for longevity in which effects of these interactions were stronger in females. Other traits measured, including egg-to-adult development time and viability and desiccation resistance, were unaffected by variation in mitonuclear genotype (Jelic *et al.* 2015). These results are thus somewhat consistent with theoretical prediction (including evidence of sex-specific effects on juvenile viability) and indicate some capacity for functional mtDNA polymorphisms to be maintained within populations via mitonuclear fitness interactions. Future studies that test whether or not these interactions extend to affecting sex-specific adult reproductive fitness in this species would provide valuable insights into theory on cytonuclear dynamics.

More broadly, these insights from *D. subobscura* provide motivation for renewed investigation of the capacity for mitonuclear interactions to shape fitness within populations of other species, including *D. melanogaster*. Ideally, future studies at this intrapopulation scale should leverage mtDNA haplotypes that are known to segregate at intermediate frequencies within the same populations and ensure that all genotypes under study are fully replicated to ensure that genotypic variance can be unambiguously partitioned from other sources of environmental and residual variance. Indeed, 1 recent study has emerged, suggesting capacity for intrapopulation mitonuclear interactions to affect fitness in *D. melanogaster*. Bevers *et al*. (2019) screened for mitonuclear imbalance (gene ratio distortions) across 169 lines of the *Drosophila Genetic Reference Panel* (DGRP), all of which derive from the 1 collection site in North Carolina, USA (Mackay *et al.* 2012). They identified 12 mtDNA haplotypes across these lines and close to 2,000 cases of mitonuclear allelic imbalances (large regions of the nuclear genome that were associated with genotype ratio distortions for a particular mtDNA haplotype), suggesting of mitonuclear epistasis for fitness, albeit subsequent fitness assays on mitonuclear lines created via reciprocal backcrossing of these lines did not reveal evidence for mitonuclear incompatibilities affecting development times or climbing ability of flies. Association analyses to test for mitonuclear interactions on 29 fitness phenotypes available for these lines detected associations between mitonuclear allelic combinations and starvation resistance and chill coma recovery in both sexes and old-age locomotion and paraquat resistance in females. An additional mitochondrial genome-wide association study identified 12 phenotypes that differed in expression between at least 1 pair of haplotypes, and subsequent experiments directly linked mtDNA haplotype to food intake rates of the lines (Bevers *et al.* 2019).

## Mitochondrial variation, mitonuclear interactions, and life history—interpopulation (intraspecific) and interspecific tests

The theoretical work modeling conditions under which cytonuclear interactions would uphold polymorphisms across interacting cytoplasmic and nuclear loci inspired further empirical tests of cytonuclear interactions across broader biological scales, those in which genetic strains were generated in which the interacting cytoplasmic and nuclear genotypes were sourced from distinct genetic lineages (either different populations of the same species or from closely related congeneric species). Many of these studies have been conducted in *Drosophila*. Early studies to take this approach included those by Clark (1985) and Hiraizumi (1985), each of which showed cytonuclear interactions affecting components of juvenile (via an assay of chromosome segregation) and adult female fitness (via an assay of adult female fecundity) in *D. melanogaster* (Clark 1985; Hiraizumi 1985). The results of these studies, when reconciled with previous empirical tests conducted at the intrapopulation level, suggested that while there may be limited capacity for fitness-modifying polymorphisms across interacting cytoplasmic and nuclear genotypes to be maintained within populations, there was indeed greater scope for the cytoplasmic genotypes of distinct lineages to diverge molecularly across functional loci and ultimately lead to cytonuclear fitness interactions in cases where the distinct lineages would come into secondary contact. These studies subsequently motivated numerous other studies over the next decades that sought to further explore the capacity of the mitochondrial genome to harbor phenotype-modifying genetic variation, via either additive mitochondrial haplotype effects or mitonuclear interactions on bioenergetics, metabolic rate, and components of life history. We review this literature below.

### Studies from *D. subobscura*

Christie *et al*. (2004) measured egg–larvae and larvae–adult viabilities and developmental times, longevity, and resistance to desiccation in isofemale lines drawn from distinct populations of *D. subobscura* bearing haplotypes I or II (Christie *et al.* 2004). Their findings revealed a higher net fitness of haplotype II across surveyed fitness traits. Work on the same populations of flies revealed an intriguing pattern of assortative mating where males and females bearing haplotype I mated together more often than with flies of the other haplotype, suggestive of a link between mitochondrial haplotype and precopulatory behavior. These patterns were, however, not replicable when flies of the same 2 haplotypes were drawn directly from a wild population (Castro *et al.* 2003).

In another series of experiments, Christie *et al.* (2011) introgressed eight *D. subobscura* haplotypes (I–XIII) taken from a solitary wild population into an identical common coevolved nuclear background. The authors also included 1 haplotype (VIII) sourced from a geographically isolated island, which was also introgressed into the common nuclear background, as well as its own coevolved nuclear background. The authors then examined haplotype-specific differences in larvae–adult development time, fertility, and longevity across these strains. The experiments revealed significant differences across haplotypes in all measured traits, although in contrast to previous findings (Christie *et al.* 2004), these effects were not mediated by pairwise differences between haplotypes I and II. The haplotype effects remained when removing haplotype VIII from the analyses, reinforcing the capacity for populations to harbor multiple diverse mtDNA haplotypes that confer functional effects on fitness (Jelic *et al.* 2015; Kurbalija Novičić *et al.* 2015). While it is unclear why the study by Christie *et al*. (2011) came to a different conclusion than that of Christie *et al*. (2004) with respect to the effects of haplotypes I and II, these discrepancies may well result from differences in nuclear backgrounds used in each study, particularly when noting the mitonuclear interactions detected by Jelic *et al*. (2015) in their intrapopulation study of the same species.

### Studies from *Drosophila simulans*

Researchers have probed the functional significance of mitochondrial genetic variation in *D. simulans*. The species is characterized by 3 main global haplotypes—*si*I, *si*II, and *si*III—that diverge from each other at ∼2.5% of nucleotide positions. James and Ballard (2003) sampled 3 strains of fly, each carrying a different haplotype, and then introgressed the haplotypes of each into the nuclear backgrounds of the others, in replicate. They identified effects of mitochondrial haplotype, and mitonuclear interactions, on larval development times and adult male survival, but not male activity. Flies with the *s*I haplotype exhibited not only the fastest development times but also the lowest probability of surviving, suggestive of a life-history trade-off linked to the mtDNA, albeit the haplotype effects were contingent on the nuclear background in which they were expressed for each trait. In a subsequent series of experiments, comparisons between 8 isofemale lines bearing either the *si*II or *si*III haplotype indicated that those harboring *si*II mtDNA had lower cytochrome c oxidase activity and lower starvation resistance, but greater egg size, fecundity, and recovered faster from chill coma than flies with *si*III haplotypes (Ballard *et al.* 2007). However, because mitochondrial haplotypes remained expressed alongside their native nuclear backgrounds in that study, it is likely that these observed differences were influenced by the nuclear genotypic differences that delineated each strain, and it was not possible to statistically partition out the effect of the mtDNA haplotypes from the nuclear background.

Other studies have sought to explore the proximate basis of the mitochondrial haplotype effects on fitness in *D. simulans*, by testing for differences in bioenergetic function of the mETS complexes of the mitochondria across haplotypes *si*I, *si*II, and *si*III. The results of these studies have generally differed according to the methodology used (Sackton *et al.* 2003; Ballard *et al.* 2007; Katewa and Ballard 2007; Pichaud *et al.* 2010, 2011, 2012). Initially conducted on isolated mitochondria, and later in permeabilized fibers of flight muscle, these studies determined thermal sensitivities and enzymatic capacities of the mETS componentry across combinations of cytonuclear genotype in which *D. simulans* haplotypes were either placed alongside various nuclear genotypic backgrounds sourced from the same species (intraspecific combinations) or alongside a nuclear background of the congeneric *Drosophila mauritiana* (interspecific combination). Disruption of the cytonuclear lineage via introgression of haplotypes *si*I and *si*II into the nuclear background of *D. mauritiana* revealed a decrease in COX activity in crude fly homogenates (Sackton *et al.* 2003), a result in line with a prediction that mtDNA and nuclear-encoded subunits of the mETS may coevolve and that disruption of these coevolved subunits, through interspecies cytonuclear substitution, would result in decreased phenotypic performance (Rand *et al.* 2004).

Further comparisons of mitochondrial respiration, proton leak, and electron flux of the mETC across males drawn from isofemale lineages of *D. simulans* bearing either the *si*II or *si*III haplotype have suggested bioenergetic differences associated with each haplotype. However, while analyses based on assays of isolated mitochondria have revealed superior metabolic performance of individuals harboring the *si*III haplotype (Ballard *et al.* 2007; Katewa and Ballard 2007), the relative performance of each haplotype is sensitive to the temperature at which the flies are reared (Pichaud *et al.* 2010), indicating the potential for involvement of genetic variation in the mitogenome in genotype-by-environment interactions and phenotypic plasticity (reviewed in *Mitochondrial gene-by-environment interactions* section). Furthermore, analyses based on in situ analyses of mitochondrial function from permeabilized fibers of flight muscle have tended to show results contrary in direction to those that used isolate mitochondria, with increased catalytic capacity and COX activity in flies harboring the *si*II relative to *si*III haplotype (Pichaud *et al.* 2011, 2012). The differences in results between in vitro studies utilizing mitochondrial homogenates and in situ studies using permeabilized fibers raise the question of which methodological approach is more informative when measuring and making ecologically relevant inferences of mitochondrial bioenergetics. While a definitive answer to this question is not easy, most subsequent studies of bioenergetic function in *Drosophila* have focused on analyses of permeabilized fibers. While analyses of fibers enables less precision in the control of cellular conditions, they enable an ecologically relevant measurement of mitochondrial function in which the cellular network of the analyzed tissues remains preserved at the time of measurement (Kuznetsov *et al.* 2008; Koch *et al.* 2021).

### Studies from *D. melanogaster*

The literature on mitochondrial haplotype effects and mitonuclear interactions in *D. melanogaster* is extensive, and the species has become established as one of the core model systems to test questions in the field of mitochondrial evolutionary biology. Many studies have created panels of strains that comprise the mtDNA haplotypes of congeneric species from the *D. melanogaster* species subgroup (consisting of *D. melanogaster*, *simulans*, and *mauritiana* mtDNA), typically expressed alongside 1 or several distinct nuclear genetic backgrounds sourced from *D. melanogaster* (Rand *et al.* 2006; Montooth *et al.* 2009; Meiklejohn *et al.* 2013; Zhu *et al.* 2014). This interspecific approach has 2 strengths. First, by increasing the molecular divergence separating the mtDNA haplotypes under test, these studies presumably increase the effect size associated with the mitonuclear interactions, increasing the chances of detection. Furthermore, for studies that create panels of strains that comprise multiple mtDNA haplotypes per species (e.g. 2 haplotypes sourced from *D. simulans* and 2 from *D. melanogaster*, placed against *D. melanogaster* nuclear backgrounds), this approach provides a means to test whether the magnitude of effect size associated with the mitonuclear interaction is increased when the interacting mitochondrial and nuclear genotypes are sourced from divergent species (interspecific interactions) rather than from the same species (intraspecific interactions). Second, under the prediction that strong selection on the mitonuclear genotype will lead to tight intergenomic coadaptation, this approach provides an opportunity to test the prediction that disruption of species-specific mitonuclear complexes will lead to phenotypic disruption (Rand *et al.* 2004).

Other studies have created panels of strains in which the interacting mitochondrial and nuclear genotypes are all sourced from within *D. melanogaster* (Rand *et al.* 2001; Clancy 2008; Aw *et al.* 2018; Drummond *et al.* 2019; Vaught *et al.* 2020; Carnegie *et al.* 2021). There are much lower levels of nucleotide divergence across the major mtDNA haplotypes within this species (∼0.3%) than either *D. subobscura* or *D. simulans*, but notably this level of divergence is similar to the divergence separating major human mtDNA haplogroups (Morrow *et al.* 2015). This implies that results from studies that examine the phenotypic consequences of mitonuclear interactions in *D. melanogaster* may provide insights into the potential for mitonuclear interactions to shape similar evolutionary processes and patterns in humans.

We review the interspecific and intraspecific studies that have utilized *D. melanogaster* below and highlight key observations to emerge from this research.

#### Interspecific studies

Studies that have sourced mtDNA haplotypes from distinct congeneric species, placing them alongside nuclear backgrounds sourced from *D. melanogaster*, have reported that mitonuclear interactions shape life-history phenotype, through intermediary effects on mitochondrial bioenergetic function. Rand *et al.* (2006) compared longevity across *D. melanogaster* populations harboring different mtDNA haplotypes, with offspring of interpopulation reciprocal crosses of *D. melanogaster* (in which hybrid female offspring carry identical nuclear genotypes but different mtDNA haplotypes), and offspring of reciprocal crosses in which 1 member of the cross contained an mtDNA haplotype derived from *D. simulans* in a *D. melanogaster* nuclear background, and the other member contained a *D. melanogaster* mtDNA haplotype alongside *D. melanogaster* nuclear background. This design enabled the authors to test 2 predictions: first, that the magnitude of effect associated with the mitonuclear interaction will increase with the degree of molecular divergence separating the haplotypes used in each reciprocal cross; and second, that reciprocal crosses resulting in interspecific combinations of mitonuclear genotype would result in reductions in longevity relative to intraspecific combinations. The results supported the first prediction that the magnitude of mitonuclear effects increased with the degree of genetic divergence. Contrary to the second prediction, however, offspring inheriting the *D. simulans* haplotype was not consistently short-lived as might be predicted if interspecific mitonuclear combinations had resulted in disruption of coadapted mitonuclear lineages. This result is notable given that >500 nucleotide substitutions separate the mtDNA of *D. simulans* and *D. melanogaster*, implying that this level of divergence between haplotypes is insufficient to result in consistent intergenomic incompatibilities. Instead, the interspecific fly strains exhibited a large range of variation in longevity falling within that observed for the intraspecific mitonuclear combinations (Rand *et al.* 2006).

A subsequent study by Montooth *et al*. (2010) placed 9 mtDNA haplotypes from *D. melanogaster*, *D. simulans*, and *D. mauritiana* into 2 distinct *D. melanogaster* nuclear backgrounds, as well as a further set of strains in which the same mtDNA haplotypes were placed alongside 2 different *D. melanogaster* X-chromosome genotypes but otherwise standardized autosomes. The authors then measured relative male and female fitness of juveniles, via a chromosome segregation study, for the different combinations of mitonuclear genotype. The authors identified general mitonuclear interactions affecting relative juvenile fitness of females, but not males. Interspecific mitonuclear combinations did not appear to result in lower relative fitness than intraspecific combinations, with exception of 1 particular *D. simulans* (simw501) haplotype expressed against the Oregon (*OreR*) nuclear background, which was associated with both low productivity and low female relative juvenile fitness. This specific mitonuclear interaction has since been the focus on intense study (reviewed in *An interspecific mitonuclear incompatibility* section). Investigation of the mito-X chromosome combinations indicated that mito-X interactions affected male, but not female, juvenile relative fitness, but that the interspecific mito-X combinations did not result in greater effects on fitness than the intraspecific combinations (Montooth *et al.* 2010).

##### An interspecific mitonuclear incompatibility

Meiklejohn *et al*. (2013) further probed the apparent mitonuclear incompatibility involving the *D. simulans* mtDNA haplotype (simw501) and the *D. melanogaster OreR* nuclear background first reported in Montooth *et al.* (2010), investigating its effect on development and reproduction, and mapping the nucleotide polymorphisms involved in the interaction. The authors examined mitonuclear interactions across 6 combinations of mitonuclear genotype (2 different *D. simulans* haplotypes and 1 *D. melanogaster* haplotype expressed against 2 different *D. melanogaster* haplotypes). They confirmed that the interaction between *simw501* mtDNA and the *OreR* nuclear background led to delays in development from egg to adulthood of ∼2 days (through extension to larval development and time spent as pupae), decreased fecundity of females of ∼50%, and shortening of the thoracic mechanosensory bristles of adult flies. Remarkably these phenotypes were not observed when the same haplotype was placed alongside a second *D. melanogaster* nuclear background (AutW132) and were not observed for a second *simulans* haplotype in the *D. melanogaster* background. Thus, the interspecific incompatibility is mediated by polymorphisms contained to specific mtDNA and nuclear variants of each of the species of this particular cross. Sequencing of *D. simulans* mtDNA revealed a SNP specific to the *mt:tRNA^Tyr^* in the simw501 haplotype, located at the base of the anticodon stem changing a G:C (the combination that is found across all *D. melanogaster mt:tRNA^Tyr^* sequences) to a G:U. The authors used meiotic mapping to locate the nuclear polymorphism interacting with the mitochondrial tRNA to a gene encoding the aminoacyl-tRNA synthetase for tyrosine in the mitochondria, showing a solitary nonsynonymous polymorphism, which changes a conserved alanine to a valine at 1 point of the amino acid sequence of the gene, separating the 2 nuclear backgrounds. They then created transgenic strains that differed at this nonsynonymous polymorphism. Experiments on these transgenic strains confirmed that this polymorphism was mediating the mitonuclear interaction. The authors hypothesized that the incompatibility was mediated by a reduced pool of tyrosine charged tRNAs, compromising protein translation in the mitochondria. In support of this hypothesized model, assays of mtETS enzyme complexes I, III, and IV (which are jointly comprised of mitochondrial and nuclear-encoded subunits), using mitochondria isolated from homogenized adult flies of both sexes revealed decreased enzyme activities, while complex II (comprised of nuclear subunits alone) was unaffected, as was the abundance of mitochondria as indicated by levels of citrate synthase (Meiklejohn *et al.* 2013).

Subsequent studies have leveraged the same set of strains to further characterize this incompatibility. Hoekstra *et al*. (2013) confirmed longer development time, low proportion and height of pupation, and greater mass-specific metabolic rate of larvae harboring the incompatible genotype. Intriguingly, these negative effects were ameliorated at cooler temperatures (Hoekstra *et al.* 2013), a finding that has been followed by further studies revealing a complex interplay between genotype and environment in regulating phenotypic expression associated with this incompatibility (reviewed in *Mitochondrial gene by environment interactions* section). Hoekstra *et al*. (2018) reported no effects of the incompatibility on body mass nor on mass-specific metabolic rate of adult females.

Holmbeck *et al*. (2015) used the same mitonuclear and transgenic strains to confirm that the incompatibility specifically depresses activity of complexes I and IV in mated females and decreases bristle length. They also reported deficiencies in flight capacity, but there was no significant difference in climbing capacity, of the flies harboring the incompatible genotype. Investigation of the mitochondrial morphology of this genotype indicated structural differences relative to that of other genotypes—loosely packed cristae structure, evidence of swirled cristae structure that may be indicative of cristae collapse and more mitochondria per unit area (Holmbeck *et al.* 2015). Buchanan *et al.* (2018) studied life-history responses of male and female flies following infection with the natural pathogen *Providencia rettgeri*, reporting lower survival and reproductive success of female flies harboring this incompatibility relative to those of other genotypes, with these effects manifesting specifically following infection. This result suggests that this mitonuclear incompatibility may result in an energy deficiency and resulting allocation trade-off between investments into immune defense versus other components of life history (Buchanan *et al.* 2018).

Follow-up study by Matoo *et al*. (2019) of the metabolic implications for larvae harboring this incompatibility reported higher levels of citrate synthase associated with the incompatibility in larvae of all 3 instars (indicating higher mitochondrial abundance within larvae of this genotype suggestive of a compensatory response to lower enzyme activities within the mtETS). The authors also observed higher levels of accumulated reactive oxygen species (ROS) at second instar (as indicated by elevated hydrogen peroxide, suggesting the genotype is susceptible to oxidative stress) and higher levels of lactate at second instar (a by-product of anaerobic respiration via glycolysis, suggesting higher reliance of larvae of this genotype on non-OXPHOS–mediated energy production as a compensatory response) in flies with the incompatibility. Larvae with the incompatible genotype also exhibited low mitochondrial membrane potential at the second and third instar stages (Matoo *et al.* 2019).

A final study of the bioenergetics of this incompatibility in adult males, using permeabilized thoracic fiber for assays of respiratory function and isolated mitochondria for assays of ROS production, confirmed a decrease in mitochondrial oxygen consumption (particularly in mETS complex IV) in flies harboring the incompatibility (Pichaud *et al.* 2019). The study, however, revealed the signature of a compensatory response, via an approximate 300% increase in the number of mtDNA copies in mitochondria harboring the incompatible genotype. Contrary to the results of Meiklejohn *et al*. (2013), Holmbeck *et al.* (2015), and Matoo *et al.* (2019), the authors showed a concomitant reduction in citrate synthase activity in adult flies, indicative of lower abundance of mitochondria in the incompatible genotype. These results suggested that flies with the incompatible genotype had fewer mitochondria, albeit with each mitochondrion presumably loaded with many more copies of mtDNA than flies of other mitonuclear genotypes. Consistent with Matoo *et al*. (2019), the authors reported that these mitochondria exhibited 50% higher rates of hydrogen peroxide production in adults of 25 days of age, indicative of ROS overproduction and likely oxidative stress (Pichaud *et al.* 2019).

#### Intraspecific studies

Extending on early studies by Clark (1985) and Hiraizumi (1985), many studies of mitonuclear variation in *D. melanogaster* have leveraged an intraspecific approach, sampling mtDNA variation from diverse natural or laboratory populations and placing this alongside controlled or varied nuclear backgrounds drawn from the same species. Similar to their interspecific counterparts, these studies have generally revealed effects of both mtDNA haplotype variation, and mitonuclear interactions, on bioenergetic, physiological, and life-history phenotypes. We highlight key examples below.

Early work that substituted cytoplasmic and nuclear backgrounds of different *D. melanogaster* strains suggested age-dependent effects on activity level that map to the cytonuclear genotype, possibly indicative of mitonuclear interactions (Driver 2000). Further study substituting cytoplasms from long-lived strains into control strains, and *vice versa*, indicated that the long-lived phenotype mapped to the cytoplasm, with ROS production in young males negatively correlating with longevity (Driver and Tawadros 2000). A further study treated these same strains with antibiotics to remove possible cellular, and cytoplasmic-transmitted, bacteria, thus homing in on whether the cytoplasmic effects might map specifically to the mtDNA haplotype. The effects on life span reported in Driver and Tawadros (2000) generally disappeared in antibiotic treated strains, but effects of mtDNA haplotype on lipid peroxide concentrations (an index of oxidative damage) and hydrogen peroxide were reported, as well as age-related changes in behavior (Driver *et al.* 2004).

In 2008, Clancy tested for mitonuclear interactions on male longevity in *D. melanogaster*. A set of 8 different mtDNA haplotypes sourced from geographically disjunct populations across the globe was collated, and these haplotypes then placed alongside a near-isogenic nuclear background (w*^1118^*). Clancy first assayed flies of each strain for survival, revealing differences across haplotypes in their effects on survival. Clancy then investigated whether survival was affected by mitonuclear interactions, by assaying longevity of male F1 offspring generated by crosses between females of 3 of these mitochondrial strains and males of 8 distinct isofemale lines that provided target nuclear backgrounds. F1 flies thus possessed 1 haploid copy of the w*^1118^* nuclear background and a haploid copy of a target nuclear background. All strains had been cured of *Wolbachia*, enabling Clancy to home in on mitochondrial effects on longevity, and whether these effects were contingent on nuclear background. Clancy found clear mitonuclear interactions for male longevity. In particular, longevity differences between the 3 haplotypes tested were much larger on some nuclear backgrounds than others (Clancy 2008).

Using part of the same panel of strains, Aw *et al.* (2011) studied effects of 4 mtDNA haplotypes alongside the w*^1118^* nuclear background on starvation resistance, components of male reproductive success, lipid proportion, and physical activity. They found that the effects of the haplotypes on each trait differed according to whether the adult flies were assayed in early or mid-life. In particular, a haplotype sourced from Japan exhibited both the lowest starvation resistance, and the lowest lipid proportion, but also some evidence of the shallowest decline in male reproductive success with male age relative to the other haplotypes sampled (Aw *et al.* 2011). Correa *et al*. (2012) examined bioenergetic differences from permeabilized fibers sampled from adult males of the same panel of 4 haplotypes across early and mid-adult life stages. They reported that males harboring the haplotype sourced from Japan had markedly lower State 3 (Complex I phosphorylating) respiration rates and higher hydrogen peroxide production at younger age, and higher mtDNA copy number at both age classes, relative to males of other haplotypes. The authors hypothesized that the Japan haplotype carries a mutation load that results in poor mitochondrial function, leading to heightened mtDNA copy number as a compensatory response to maintain ATP production (Correa *et al.* 2012).

Subsequent studies have leveraged an expanded set of strains representing 13 haplotypes, originally collated by Clancy (2008), to investigate the association between mitochondrial genotype, physiology, and life history. Wolff, Pichaud, *et al.* (2016) studied effects across 12 haplotypes—including 3 of the 4 haplotypes used by Correa *et al*. (2012)—placed alongside the same nuclear genomic background (*w^1118^*) as had been used by Correa *et al*. (2012). They measured various components of respiratory performance across the complexes of the mETS, from permeabilized fibers of males and females at 2 ages (15 and 25 days of adult age). These respiratory parameters were reduced to 2 major axes of function using principal component analysis, one of which represented respiratory rate and the other mitochondrial quantity. The authors reported effects mediated by mitochondrial haplotype on both respiratory rate and mitochondrial quantity, with the pattern of effects across haplotypes exhibiting a high degree of context dependency, across males and females, and according to the age of the flies (Wolff, Pichaud, *et al.* 2016). Notably, the results were consistent with the key results of Correa *et al*. (2012)—the Japan haplotype was associated with high mitochondrial quantity in males of both age classes and low respiratory rate at least in older males. These effects of the Japan haplotype on low male respiratory performance were specific to males.

Several other studies have leveraged the same panel as used by Wolff, Pichaud, *et al.* (2016) and examined fitness consequences in both males and females, revealing sex specificity of effects (Camus *et al.* 2012; Camus and Dowling 2018), or across diverse environments, revealing mitochondrial gene by environmental interactions (Aw *et al.* 2018; Nagarajan-Radha *et al.* 2019; Camus, O’Leary, *et al.* 2020). These studies are described in the *Sex specificity of mitochondrial haplotype effects* and *Mitochondrial gene by environment interactions* sections below.

Salminen *et al.* (2017) created a similar set of genetic strains, introgressing 10 mtDNA haplotypes into a standard nuclear background (derived from Oregon RT) and also backcrossing 4 of the 10 haplotypes into a second nuclear background. The authors then examined haplotype effects on mtDNA copy number, egg-to-adult development time, juvenile body weight in both nuclear backgrounds, and oxygen consumption and activity and formation of the respiratory complexes in 1 nuclear background. They reported mtDNA haplotype effects on mtDNA copy number, which seemed largely independent of nuclear background, and haplotype effects on development time and juvenile body weight. Whether or not the effects of haplotype interacted with the nuclear background was difficult to discern, given the unbalanced nature of the data set (10 haplotypes in 1 nuclear background and 4 haplotypes in the other) and given that the statistical tests for mitonuclear interactions were not reported in the manuscript. However, the authors did report interesting correlations between mtDNA copy number and juvenile body weight and copy number and egg-to-adult development time across haplotypes, indicating that haplotypes associated with lower copy number were associated with faster development to adulthood in both males and females and heavier juvenile body mass in females, in the Oregon RT nuclear background. They also reported haplotype effects on mitochondrial substrate oxidation of OXPHOS complexes I and III, in which haplotypes associated with lower copy number exhibited heightened oxidation of complex I and III substrates, and this appeared linked to elevated formation of supercomplexes (supramolecular structures) manifesting complex I activity, indicating a higher order arrangement of the OXPHOS complexes in these haplotypes (Salminen *et al.* 2017).

Motivated by a series of studies that implicated mtDNA sequence variation in the dynamics of climatic adaptation, by revealing correlations between mutational patterns of human mtDNA haplogroups and climatic zones (Mishmar *et al.* 2003; Ruiz-Pesini *et al.* 2004; Balloux *et al.* 2009), Camus *et al.* (2017) investigated spatial distributions of mtDNA haplotypes in natural populations of *D. melanogaster* in Australia, testing 2 predictions. The first prediction was that frequencies of mtDNA haplotypes would exhibit latitudinal clines, with some haplotypes predominating at higher frequency in low-latitudinal, subtropical localities, and others predominating in high-latitudinal, temperate localities. The second prediction was that spatial distributions of haplotypes would correspond to the direct effects of these haplotypes on the thermal tolerance phenotypes of flies, with haplotypes that are at higher population frequencies at low latitudes conferring higher tolerance to heat stress and those at higher population frequencies at high latitudes higher tolerance to cold stress. The authors identified 2 main groups of haplotypes segregating along the Australian eastern seaboard, each characterized by 1 major haplotype, which were delineated by 15 polymorphisms. The haplotypes exhibited a latitudinal cline, with the A1 haplotype predominating in lower latitude populations and the B1 haplotype at higher latitudes. The authors then created genetic strains in which each haplotype was placed alongside a standardized nuclear background and then characterized each strain for its capacity to confer tolerance to both extreme heat and extreme cold stress. Consistent with predictions, the A1 haplotype conferred higher tolerance to heat stress but lower tolerance to cold stress than B1 haplotypes. Thus, the thermal tolerances of the haplotypes corresponded to their spatial distributions in natural populations, consistent with the hypothesis that climatic selection had shaped the distributions of these haplotypes in the Australian fruit fly population (Camus *et al.* 2017).

#### Sex specificity of mitochondrial haplotype effects

Several studies to have examined mitonuclear and mitochondrial genetic effects on life-history expression have reported effects that are larger in magnitude in males than females (Sackton *et al.* 2003; Christie *et al.* 2011; Camus *et al.* 2012; Kurbalija Novičić *et al.* 2015; Drummond *et al.* 2019). The cause for the sex specificity has been hypothesized to lie in the mode with which mitochondrial genomes are inherited— with few exceptions, maternally (Birky 1978; but see: White *et al.* 2008). The consequence of maternal inheritance of mitochondria is that males represent an evolutionary dead end for mitochondrial genomes; as such, adaptive evolutionary responses to selection on the mitochondrial genome have generally been presumed to proceed only through females (Frank and Hurst 1996; Gemmell *et al.* 2004; Zeh and Zeh 2005; Beekman *et al.* 2014). In theory, this asymmetry in selection on mtDNA across the sexes should pose no problem for traits with identical function across both sexes, since selection on the mtDNA sequence for variants that confer optimal bioenergetic function of these traits in females should similarly confer optimal function in males. However, for sexually dimorphic or antagonistic traits, female-limited selection may in theory lead to selection of mitochondrial allelic variants that optimize bioenergetic function associated with female trait homologs, even when these same variants exert a cost to function of male homologs. This may result in the accumulation of variants in populations that are deleterious to males but beneficial, neutral, or only slightly deleterious to females (Frank and Hurst 1996; Gemmell *et al.* 2004; Beekman *et al.* 2014), a phenomenon often referred to as the *Mother's Curse* hypothesis (Gemmell *et al.* 2004). While male-harming mtDNA mutations commonly exist in plants, via cytoplasmic male sterility effects (Chase 2007), examples from animals remained elusive until recently. Notwithstanding, over the past decade, there have been numerous reports of sex specificity in the effects of mitochondrial genotype on phenotype that are consistent with predictions of the Mother's Curse hypothesis (Beekman *et al.* 2014; Vaught and Dowling 2018; Dowling and Adrian 2019), with intriguing insights coming from examples of mtDNA mutations in *D. melanogaster* that are associated with male-only sub-fertility or in-fertility, whereby females remain fully fertile (Xu *et al.* 2008; Clancy *et al.* 2011; Yee *et al.* 2013; Patel *et al.* 2016; Camus and Dowling 2018).

In particular, 2 examples have been reported of single mutations that were naturally segregating in field or laboratory populations of flies at the time of identification, in the mitochondrial cytochrome b gene (an alanine to threonine substitution at site 278 in the amino acid sequence of *mt:Cytb*, which is found on a haplotype originally collected in Brownsville, TX, USA) and cytochrome c oxidase II gene (a glycine to serine substitution at position 177 in COII, found segregating in the *w^1118^* laboratory stock), which can perturb sperm development and function leading to male sterility or subfertility while leaving female fertility intact (Clancy *et al.* 2011; Yee *et al.* 2013; Patel *et al.* 2016; Camus and Dowling 2018). In the case of each mutation, the magnitude of the male infertility impairment varies across nuclear genetic backgrounds, with the effects of the *mt:Cytb* mutation generally more severe than the *mt:COII* mutation and causing complete male sterility in certain nuclear backgrounds (Clancy *et al.* 2011; Yee *et al.* 2013; Dowling *et al.* 2015; Patel *et al.* 2016; Wolff, Tompkins, *et al.* 2016; Camus and Dowling 2018).

Remarkably, the effects of the Brownsville haplotype (which harbors the *mt:Cytb* mutation) on bioenergetic function have been shown to be sex specific; males at 15 and 25 days of adult age suffer reduced OXPHOS respiratory capacity relative to other haplotypes and generally low mass-specific metabolic rate, while bioenergetic and metabolic function in females carrying this haplotype is normal. Furthermore, males that carry this haplotype are characterized by lower levels of citrate synthase (an indicator of mitochondrial quantity) present in permeabilized wing fibers than males harboring other haplotypes, while females with this haplotype exhibit normal mitochondrial quantities (Wolff, Pichaud, *et al.* 2016; Nagarajan-Radha *et al.* 2020). Yet, it appears to be only male reproductive outcomes that are compromised by this bioenergetic deficiency. Males that carry the Brownsville haplotype have been reported to enjoy normal or even longer lives than males carrying other haplotypes, whereas females despite maintaining high fecundity appear to suffer from reduced survival (Camus *et al.* 2012, 2015; Nagarajan-Radha *et al.* 2019). Juvenile flies of both sexes that carry the Brownsville mutation exhibit faster development to adulthood, and pupal viability, than flies of other haplotypes (Wolff, Tompkins, *et al.* 2016; Camus and Dowling 2018). The molecular mechanisms that regulate sex specificity of this mutation remain elusive, although both females and males that carry the *mt:Cytb* mutation found in the Brownville haplotype were reported to exhibit depressed expression of the cytochrome b gene (Camus *et al.* 2015). Unlike the case of the *mt:Cytb* mutation described above, the effects of the *mt:COII* mutation on bioenergetic function are shared across the sexes, with impairment of cytochrome c oxidase activity in males and females when flies were reared at 29°C. Furthermore, male and female flies carrying the *mt:COII* mutation do not exhibit any differences in survival relative to their wild-type counterparts, suggesting that, unlike the *mt:Cytb* mutation, this mutation does not confer trade-offs between male reproductive fertility and life span (Patel *et al.* 2016).

That the male fertility impairment associated with the *mt:Cytb* and *mt:COII* mutations can be partially restored, or even fully restored in the case of the *mt:COII* mutation, when placed alongside certain nuclear genetic backgrounds, suggests the presence of compensatory restorer alleles in the nuclear genome that regulate the phenotypic effects of mtDNA mutations (Dowling *et al.* 2015; Patel *et al.* 2016; Wolff, Tompkins, *et al.* 2016). Because of its male specificity in inheritance and gene content (Bachtrog 2013), the Y chromosome has been hypothesized as a candidate genomic region to evolve compensatory restorer alleles, since Y-linked restorer alleles would have capacity to moderate effects of male-harming mitochondria without interfering with female physiology (Rogell *et al.* 2014; Dean *et al.* 2015; Yee *et al.* 2015). This leads to the prediction that population specific (hence presumably coadapted combinations of mtDNA haplotype and Y chromosome) would confer heightened performance than disrupted combinations. Yet, although interactions between mtDNA and Y-chromosome polymorphisms have been shown to affect male mating outcomes in *D. melanogaster*, the direction of these interactions was not consistent with the predicted pattern in which disrupted combinations of mtDNA and Y genotype performed worse than population-specific combinations (Yee *et al.* 2015). Furthermore, studies have screened for mito-Y interactions affecting other traits but have found no evidence that epistasis between mtDNA and Y chromosomes mediates outcomes of longevity or locomotory activity (Dean *et al.* 2015; Ågren *et al.* 2020). Notwithstanding, studies that have examined the effects of Y chromosome, and mtDNA haplotype, substitution on genome-wide patterns of transcript expression in *D. melanogaster*, have presented evidence of a large number of nuclear genes, particularly those tied to male fertility, whose expression is co-regulated both by sequence variation in mtDNA and the Y chromosome (Rogell *et al.* 2014; Ågren *et al.* 2020).

Evidence for the Mother's Curse hypothesis in *D. melanogaster* extends beyond the example of specific mutations exhibiting sex-specific effects. Studies utilizing *D. melanogaster* have generally tested 2 different predictions of the hypothesis, corresponding to *weak* and *strong* forms of the hypothesis respectively (Dowling and Adrian 2019; Havird *et al.* 2019). The weak form of the hypothesis describes a population genetic model presented by Frank and Hurst (1996), who demonstrated that maternal inheritance of mitochondria may, in theory, facilitate the accumulation to appreciable frequencies of mtDNA mutations that are male harmful, when the same mutations are benign or only slightly detrimental in females. These mutations would accumulate under mutation–selection balance, with some becoming fixed under drift or genetic draft, and result in the accumulation of a male-biased mutation load within the mitochondrial genomes of populations. The signature of this mutation load could be experimentally detected by sampling mtDNA haplotypes from within a distribution of a species, placing these alongside a standardised and novel nuclear genetic background that lacks coevolved nuclear restorer alleles (hence exposing the effects of the male-harming mutations), and then screening for associated effects of these haplotypes on male and female phenotypes. The key prediction under this weak form of the hypothesis is that mitochondrial haplotype effects on trait expression will be larger in magnitude in males than females (Dowling and Adrian 2019). The strong form of the Mother's Curse hypothesis assumes capacity for mtDNA mutations that affect key sexually dimorphic traits to confer sexually antagonistic effects on trait expression, benefiting females but harming males. Population genetic theory has demonstrated that if such mutations occur within the mitochondrial genome, then they should come under direct positive selection in females and drive to fixation within populations despite their costs to males, at least in outbred populations (Unckless and Herren 2009). If so, this should lead to the presence of negative genetic correlations, across mtDNA haplotypes, for performance in each of the sexes; haplotypes associated with high relative performance in females should be associated with low relative performance in males (Dowling and Adrian 2019).

Studies have tested these predictions, with several utilizing the intraspecific panel of haplotypes first utilized by Clancy (2008), with 13 haplotypes sourced from different global locations placed alongside *w^1118^* nuclear background, each in independent replicate. These have generally found results that correspond with the weak form prediction, a strong bias in the magnitude of mitochondrial haplotype effect on longevity, metabolic rate, components of body size, quantity of mitochondria in thorax muscle of young flies, ROS production, mtDNA copy number, and activity of superoxidase dismutase (Camus *et al.* 2012; Wolff, Pichaud, *et al.* 2016; Aw *et al.* 2017; Drummond *et al.* 2019; Nagarajan-Radha *et al.* 2020), although these male biases are not apparent in underlying efficiency of the respiratory complexes (Wolff, Pichaud, *et al.* 2016; Aw *et al*. 2017).

Notably, a study by Carnegie *et al*. (2021), using a panel of strains represented by 9 distinct mtDNA haplotypes expressed alongside 9 nuclear backgrounds, showed that the male bias in mtDNA haplotype effect on wing size was upheld across 8 of 9 different nuclear backgrounds. Another study by Vaught *et al*. (2020) reported evidence of strong sex specificity in the outcomes of cytonuclear interactions for longevity, when cytoplasms from 3 different mass-bred populations of flies were substituted into each of the 3 populations, in all combinations. Although there was no apparent signature of male bias in the magnitude of cytoplasmic effects across all populations, 1 of the 3 populations used, sampled from a mid-latitudinal location in Australia, carried *Wolbachia*, rendering it difficult to partition mitochondrial genotypic effects from other sources of cytoplasmic variance. Notwithstanding, analysis of the mitochondrial haplotype effect within the population infected with *Wolbachia* revealed a signature of male bias in effects of 2 haplotypes segregating in that population on longevity—flies that carried the A1 haplotype outlived flies carrying the B1 haplotype, with this effect larger in males than females (Vaught *et al.* 2020). These are the same haplotypes that were previously screened for thermal tolerance by Camus *et al*. (2017), in a different nuclear background. In that study, the authors identified 1 particular sub-haplotype of B1 (which differed by 1 extra SNP, at a synonymous site in *mt:ND4*, from other B1 subhaplotypes) in which resilience to heat stress was much reduced in males but not females, relative to other B1 sub-haplotypes (Camus *et al.* 2017).

A transcriptomic study by Innocenti *et al.* (2011) was particularly noteworthy in terms of the degree of male bias reported, with the authors reporting that the identity of the mtDNA haplotype (across 5 haplotypes of the panel first used by Clancy, 2008) affected the expression of over 1,000 nuclear genes in the male fly but just a tiny handful of genes in the female fly, with many of the differentially expressed genes enriched in the male reproductive tissues (testes and accessory glands) and previously identified to be important in the modulation of male reproductive outcomes. These results were supported by another recent transcriptomic study by Ågren *et al.* (2020), which assessed genome-wide patterns of differential transcript expression across 6 mtDNA haplotypes of *D. melanogaster*. Although the study examined transcriptomic responses in males only, consistent with Innocenti *et al*. (2011), they observed differential expression of 760 genes, including genes related to male fertility that exhibit elevated expression in testes and accessory glands (Ågren *et al.* 2020).

Other studies utilizing the same intraspecific panel of haplotypes that revealed male-biased effects on longevity, genome-wide transcript expression, and metabolic rate of flies (Innocenti *et al.* 2011; Camus *et al.* 2012; Nagarajan-Radha *et al.* 2020) also reported effects consistent with the strong form prediction for components of reproductive success and metabolic rate (Camus and Dowling 2018; Nagarajan-Radha *et al.* 2020). In each case, negative intersexual correlations were reported across mtDNA haplotypes for these traits, with the haplotypes associated with high relative trait expression in 1 sex associated with low relative trait expression in the other. In the case of metabolic rate, Nagarajan-Radha *et al*. (2020) furthermore identified male-specific patterns of antagonistic pleiotropy across haplotypes, in which haplotypes associated with high relative metabolic rate in males were associated with shorter male longevity. Consistent with these findings, Rand *et al*. (2001) identified signatures of sexual antagonism of cytoplasmic strains, and mtDNA haplotypes, on relative juvenile fitness, as measured using a chromosome segregation assay. Currently, the relative contribution of weak form versus strong form mutations to shaping sex specificity of effects linked to mitochondrial haplotype variation remains unclear and represents a challenge for future research to address.

Notably, while numerous studies have provided support for the weak and strong forms of Mother's Curse in *D. melanogaster*, other studies have not. In particular, studies that have utilized interspecific panels of mitonuclear genotypes to test for sex specificity of effects, through substitution of mtDNA from *D. simulans* into nuclear backgrounds from *D. melanogaster*, have generally failed to observe patterns predicted under the weak form of the Mother's Curse. Two studies examining components of juvenile fitness (egg-to-adult development time or chromosome segregation assays) reported a lack of general male bias in effects (Montooth *et al.* 2010; Mossman, Biancani, *et al.* 2016). As outlined in the *Interspecific studies* section, Montooth *et al*. (2010) reported female-specific effects of mitonuclear interactions on juvenile fitness (inconsistent with the Mother's Curse hypothesis) but male-specific effects (consistent with the hypothesis) when the interactions were limited to those between X-linked nuclear loci and mtDNA haplotype. Mossman, Biancani, *et al.* (2016) investigated mitonuclear interactions for development time across a panel of 72 mitonuclear genotypes (6 haplotypes, 3 each from *D. melanogaster* and *D. simulans* placed alongside nuclear backgrounds sourced from 12 DGRP lines), reporting no statistically significant mtDNA- or mitonuclear-mediated effects on sex differences in development time or sex ratio of egg clutches, albeit not formally testing for statistical interactions between mtDNA and sex or mDNA, nuclear genotype, and sex on development time or sex ratio.

In interspecific studies testing the role of mitochondrial and nuclear variation in the regulation of genome-wide patterns of transcript expression, results have been mixed. In 1 study, Mossman, Tross, *et al.* (2016) tested the effects of 2 different haplotypes—1 from *D. melanogaster* and 1 from *D. simulans*, on genome-wide patterns of gene expression when placed alongside 2 different nuclear backgrounds from *D. melanogaster*. The authors reported generally larger effects of mtDNA substitution on patterns of differential expression in females than in males. Notably, interspecies substitution of mtDNA (*D. simulans* mtDNA in *D. melanogaster* nuclear backgrounds) tended to result in downregulation of mtDNA-encoded OXPHOS genes, with the exception of *mt:ND2*, which was upregulated (Mossman, Tross, *et al.* 2016). In a subsequent study, Mossman, Biancani, *et al.* (2019) utilized a larger panel of 4 mtDNA haplotypes (2 from *D. simulans* and 2 from *D. melanogaster*), placed alongside 2 different *D. melanogaster* nuclear backgrounds, reporting effects of mtDNA haplotype on the expression of numerous genes, with no apparent sex bias. The authors further partitioned the analyses into effects of mtDNA haplotypes in general (effects of all 4 haplotypes on nuclear expression patterns) and effects at the level of the 2 species (effects of *D. simulans* haplotypes vs. effects of *D. melanogaster*). Notably, and consistent with the results of the study of Mossman, Tross, *et al.* (2016), numbers of differentially expressed genes at the species-level haplotype comparison were much more strongly female biased, than numbers at the level of comparison of the 4 haplotypes (Mossman, Biancani, *et al.* 2019).

The general discrepancy in the direction and strength of sex specificity between results of studies conducted at the intraspecific scale, and those at the interspecific scale, is intriguing. Dowling and Adrian (2019) discussed possible reasons for discrepancies in detail and suggested that analyses of mtDNA haplotype effects that utilize interspecies mtDNA substitution might uncover cryptic but nonadaptive, nuclear genetic variation that is typically masked from expression in the intraspecies context; that is, mtDNA polymorphisms that were honed by selection within the nuclear environment of the species in which they evolved may no longer be adaptive within the divergent nuclear backgrounds of the congener species. In this context, Dowling and Adrian (2019) suggested that the intraspecific context is the appropriate scale to test predictions of Mother's Curse, while also noting caution should be taken to avoid the potential for inferences to be affected by sampling error, in the case of studies in which the number of levels of the factor of key interest (the mtDNA haplotype) was low.

Finally, we note 1 other set of studies that has sought to test a third prediction that derives from the Mother's Curse hypothesis. The prediction is that experimental disruption of putatively coevolved combinations of mitochondrial and nuclear genotype will lead to decreases in fitness, with the extent of fitness decrease being larger in males than females. The prediction rests on the assumption that mitochondrial haplotypes will accumulate mutations, which select for restorer adaptations in the nuclear genome that offset mtDNA-incurred fitness losses. Given maternal transmission of mtDNA, males will carry an extra load of male-harming mtDNA mutations, which are neutral or near neutral in females, which will select for their own specific set of restorer mutations. As such, disruption of population- or species-specific combinations of mitonuclear genotype should lead to depressed fitness, but the extent of the fitness depression should be greater in males than females (Dowling and Adrian 2019). Consistent with this prediction, 1 early study by Sackton *et al.* (2003) found that activity levels of the mitochondrial enzyme, cytochrome c oxidase, were lower for male but not female, flies carrying disrupted interspecific combinations of *D. simulans* mtDNA haplotypes expressed against *D. mauritiana* nuclear backgrounds. However, other studies to test the performance of interspecific combinations of *D. simulans* and *D. melanogaster* mtDNA haplotypes, expressed alongside *D. melanogaster* nuclear backgrounds, have failed to provide consistent evidence that the interspecific mitonuclear combination performs more poorly than the intraspecific combination in either sex (Rand *et al.* 2006; Montooth *et al.* 2010; Zhu *et al.* 2014; Mossman, Biancani, *et al.* 2016; Montooth *et al.* 2019).

#### Mitochondrial gene-by-environment interactions

Gene-by-environment interactions are pervasive in nature and refer to instances in which levels of gene expression, and consequently phenotype expression, are contingent on the particular environment in which a gene is expressed. As such, levels of phenotypically plasticity typically differ according to the nuclear genotype of the organism (Grishkevich and Yanai 2013). Studies emerging from the field of mitochondrial evolutionary genetics clearly demonstrate that mitochondrial genes are similarly sensitive to the environments in which they are expressed (Hoekstra *et al.* 2013; Pichaud *et al.* 2013; Zhu *et al.* 2014; Dean *et al.* 2015; Mossman, Biancani, *et al.* 2016; Patel *et al.* 2016; Wolff, Tompkins, *et al.* 2016).

Several studies have tested for dietary-mediated plasticity of mitochondrial haplotype effects and mitonuclear interactions. Zhu *et al*. (2014) created a set of 18 mitonuclear genotypes, substituting 5 mtDNA haplotypes (3 from *D. simulans* and 2 from *D. melanogaster*) into 3 nuclear backgrounds of *D. melanogaster*, one of which was daughterless GAL4 driver, which could be crossed to a UAS-SIR2 nuclear background or control, to achieve overexpression of SIR2, which is a family of signaling proteins involved in metabolic regulation. They then screened these genotypes across 5 different diets that differed in their yeast and sugar concentrations, reporting extensive mitochondrial haplotype-by-diet interactions and mitochondrial-by-nuclear-by-diet interactions for life span and providing evidence that the effect of SIR2 on longevity changes across mtDNA haplotypes (Zhu *et al.* 2014). Mossman, Biancani, *et al.* (2016) extended this work, measuring development time across a panel of 72 mitonuclear genotypes (3 *D. melanogaster* haplotypes, 3 *D. simulans* haplotypes, and 12 *D. melanogaster* nuclear backgrounds), when assayed on 4 diets differing in protein-to-carbohydrate ratios. They reported that outcomes of mitonuclear interactions on development time were contingent on the diets in which the flies were assayed (Mossman, Biancani, *et al.* 2016). Similar effects of the dietary environment on the expression of mitochondrial haplotype on phenotype have been observed across additional intraspecific panels of strains, moderating the expression of longevity, OXPHOS function, physical activity, and male reproductive success (Pichaud *et al.* 2013; Dean *et al.* 2015; Nagarajan-Radha *et al.* 2019; Camus, Moore, *et al.* 2020; Camus, O’Leary, *et al.* 2020; Rodríguez *et al.* 2021). For example, previously observed male biases in effects of mtDNA haplotype on longevity across a set of 13 mtDNA haplotypes from *D. melanogaster* were eroded when flies were assayed on synthetic diets of low and high protein-to-carbohydrate ratio, although the magnitude of mtDNA haplotype by diet interaction (i.e. the sensitivity of mtDNA haplotypes to dietary variation) was larger in males than females (Nagarajan-Radha *et al.* 2019). Similar results were reported by Aw *et al.* (2017), who reported that the magnitude of mtDNA by diet interaction, across 2 haplotypes and 2 diets, was larger in males than females for both fecundity and survival. These studies by Aw *et al.* (2017) and Nagarajan-Radha *et al.* (2019) suggest that males may be more sensitive than females to mitochondrial genotype by environments interaction (mtDNA-mediated plasticity).

Further evidence for dietary-mediated plasticity of mitochondrial haplotype effects comes from Camus, O’Leary, *et al.* (2020), who screened for mitonuclear interactions for survival and fecundity across 2 dietary treatments differing in protein-to-carbohydrate ratios. They reported interactions between mtDNA haplotype and sex and also between mtDNA haplotype and nuclear genotype for survival, each of which was moderated by dietary treatment, as well as an interaction between mtDNA haplotype and diet shaping female fecundity (Camus, O’Leary, *et al.* 2020). And in a study focused on the effects of diet in moderating the link between mtDNA haplotype on male reproductive success, Camus, Moore, *et al.* (2020) created 4 synthetic liquid diets differing in protein-to-carbohydrate ratio and then measured male reproductive output under noncompetitive conditions across 4 haplotypes. They revealed significant variation in both levels of consumption of the diets for males harboring different mtDNA haplotypes and also variation in the effects of diet on male reproductive success across haplotypes. In sum, dietary-mediated plasticity of effects of mtDNA haplotype on trait expression appears to be pervasive in *D. melanogaster*.

Other studies have tested for thermal plasticity of mtDNA haplotype effects on phenotype, across interspecific and intraspecific contexts, reporting mitochondrial haplotype by temperature effects on several traits. For example, the incompatibility between *D. simulans* mtDNA haplotype (simw501) and the *D. melanogaster OreR* nuclear background, highlighted in *An interspecific mitonuclear incompatibility* section, is temperature sensitive in its effects. The negative effects of the incompatibility are reduced when juveniles develop at 16°C and increased when juveniles develop at warmer temperatures. Moreover, larval metabolic rate is normal at 16°C but elevated relative to other mitonuclear genotypes at 25°C, while metabolic plasticity of larvae is reduced at cooler temperatures (Hoekstra *et al.* 2013). Subsequent research by Hoekstra *et al*. (2018) found that exposure to constant light stress influenced the severity of effects of the mitonuclear incompatibility, resulting in developmental delays, in a way that mimicked the effects of increased developmental temperature. They also reported that adult mass-corrected metabolic rate was less sensitive to mitonuclear variation, and to genotype-by-temperature interactions than was larval metabolic rate (Hoekstra *et al.* 2018), while other studies reported that unlike the case in females in which negative effects were general across temperatures, the negative effects of the mitonuclear incompatibility on male adult fitness were temperature sensitive and revealed only when males developed at warmer (28°C) temperatures, causing male sterility (Hoekstra *et al.* 2013; Montooth *et al.* 2019; Zhang *et al.* 2017). Notably, some male fertility was partially restored at this temperature when males were administered diets with increasing protein-to-carbohydrate ratios (Montooth *et al.* 2019).

Thermal plasticity of mtDNA haplotype effects has also been shown to affect expression of haplotypes in intraspecific studies in *D. melanogaster*, including haplotypes that carry the male fertility-impairing *mt:COII* and *mt:Cytb* mutations outlined in the *Sex specificity of mitochondrial haplotype effects* section (Patel *et al.* 2016; Wolff, Tompkins, *et al.* 2016). The rapidly emerging evidence for dietary and thermal plasticity of mitochondrial haplotype effects, and mitonuclear interactions, on host phenotypes, indicates a need for studies to incorporate environmental axes of variation into future studies that seek to elucidate the evolutionary and ecological implications of mitochondrial genetic variation. Now that it is clear that mitochondrial haplotype effects are sensitive to environmental heterogeneity, future studies should turn attention to the evolutionary and ecological significance of this *mtDNA-mediated plasticity*. A key question to resolve, going forward, is whether such plasticity in response to novel environments is adaptive, buffering populations from fitness losses when subjected to rapidly changing environments, or whether it is maladaptive, moving populations away from optimum fitness peaks in the new environments.

## Population cage experiments

Much of the above research has established that mtDNA haplotypes are sensitive to selection, but such studies fall short of being able to infer whether selective differences in mtDNA haplotypes can ultimately drive adaptive evolutionary change, by shaping the distribution and evolutionary trajectories of these haplotypes in natural populations. To help answer this question, population cage experiments have provided a powerful means to test the hypothesis that selection on particular mitochondrial haplotypes, or combinations of mitonuclear genotype, can lead to changes in the frequencies of segregating mtDNA haplotypes. In such experiments, large cages are seeded with populations of flies with specific starting frequencies of different mtDNA haplotypes and then monitored across generations to assess changes in haplotypes frequencies. One of the first studies that employed this design to study trajectories of mitochondrial haplotype evolution used 2 isofemale strains of *Drosophila pseudoobscura* harboring distinct mtDNA haplotypes (drawn from different subspecies—*D. pseudoobscura pseudoobscura* and *D. pseudoobscura bogotana*) to establish large fly populations with specific proportions of each haplotype (MacRae and Anderson 1988). In 1 specific population cage, 1 of the 2 haplotypes exhibited a continuous increase in frequency, starting at a frequency 30% and increasing to 77% after 5 generations, before reaching frequency equilibrium just under 80% after 6 generations (MacRae and Anderson 1988). Haplotype frequencies remained stable for the duration of the experiment (32 generations), even after perturbation of allele frequencies via addition of flies carrying the minor mtDNA haplotype at generation 22. While the perturbation at generation 22 caused a sharp 23% drop in frequency of the main haplotype, the competing haplotypes swiftly returned to equilibrium levels of ∼80% only 1 generation later. Notwithstanding, these same patterns were not observed consistently in other replicate populations initiated under the same conditions nor when the haplotypes were placed in competition with each other on a mixed nuclear background, and thus the study provided only very limited evidence that the haplotypes differ in their selective advantage (MacRae and Anderson 1988). Indeed, the haplotypes used in this study were drawn from different subspecies, which exhibit partial reproductive isolation, and it was proposed by Singh and Hale (1990) that differences in mating preferences and faster mating ability of *D. bogotana* females could have been the primary driver of the skews in mtDNA haplotype frequency in some population cages (Singh and Hale 1990).

In 1990, Fos *et al.* conducted a similar study but in *D. subobscura*. They placed each of 2 haplotypes (haplotypes I and VIII) alongside 3 different nuclear backgrounds (1 that was matched to haplotype I, 1 to haplotype VIII, and another that was a mix of each off the 2 backgrounds). A series of experiments was initiated—firstly with population cages established with 500 pairs of flies for each mtDNA haplotype at a 1:1 sex ratio and a discrete generation cycle, with 1 replicate cage for each nuclear background, and then a series of smaller culture bottles with smaller number of flies, with the mtDNA haplotypes always competing alongside the same nuclear background. By monitoring haplotype frequencies after each of 5 generations, the authors observed linear shifts in haplotype frequencies, with haplotype I exhibiting consistent increases in frequency, particularly when the experiments were conducted under larger effective population sizes (in the population cages rather than culture bottles and hence less subject to frequency changes being dictated by drift). There was however 1 exception—haplotype I decreased in frequency when competing in the nuclear background matched to haplotype VIII, suggesting that haplotypes may perform better when placed alongside their own putatively coevolved nuclear backgrounds, implicating a role for mitonuclear interactions in moderating outcomes of selection on the mtDNA haplotypes (Fos *et al.* 1990). Notwithstanding, the authors utilized just 1 population cage for each of the nuclear backgrounds, and thus it was again not possible to definitively conclude that the nuclear background had a consistent effect in shaping the trajectories of the mtDNA haplotypes across generations.

Hutter and Rand (1995) provided perhaps the strongest support for the hypothesis that selection can shape population frequencies of mtDNA haplotypes. The authors competed mtDNA variants of *D. pseudoobscura* and *Drosophila persimilis* against 1 another reciprocally in the genetic background of each species, across replicated population cages for each reciprocal combination (Hutter and Rand 1995). In these experiments, the *D. pseudoobscura* mtDNA variant increased rapidly and consistently in frequency from 50 to 80% across 4 replicate cages when expressed against its own nuclear background, indicating a strong fitness advantage of the *D. pseudoobscura* mtDNA variant over the *D. persimilis* mtDNA variant. This fitness advantage was also reflected in female fertility, with *D. pseudoobscura* females carrying their own mtDNA variant being the most fertile (Hutter and Rand 1995). This observation was consistent with that of Fos *et al.* (1990), indicating competitive superiority of mtDNA haplotypes when competing within their coevolved nuclear genetic backgrounds relative to those sourced from divergent lineages, suggesting capacity for selection on mitonuclear genotype to mediate coadaptation between genomes. In contrast, however, neither of these 2 mtDNA variants exhibited consistent changes in frequency, or differences in fertility, when expressed against the nuclear background of *D. persimilis*.

Further experiments utilizing population cages followed. In 1998, Garcίa-Martίnez *et al.* conducted a study in which they competed 2 naturally co-existing mtDNA variants (I and II) against 1 another across 4 population cages in *D. subobscura*. Results showed that when placed in competition alongside its natural heterogeneous nuclear background, haplotype II consistently reached fixation or near fixation after 13–16 generations (García-Martínez *et al.* 1998). These experiments by Garcìa-Martínez *et al.* (1998) were then extended in a comprehensive follow-up study of the competitive performance of the same haplotypes in *D. subobscura*, in which heterogeneity of the nuclear background and the level of replication were increased (Oliver *et al.* 2005). This second study additionally monitored the maintenance of chromosomal inversions (J and J_ST_) that had been reported to co-segregate in linkage disequilibrium with haplotypes I and II, to probe for nuclear factors that may impact haplotype frequencies (Oliver *et al.* 2002). However, while mitochondrial haplotype effects on fitness were detected, neither cytonuclear disequilibria nor the superiority in fitness of haplotype II over haplotype I could be confirmed. In fact, reanalysis of this data set found that changes in haplotype frequencies across generations were significantly different to those expected under scenarios of genetic drift or positive selection but consistent with expectations of negative frequency-dependent selection (Arnqvist *et al.* 2016).

Population cage studies testing for selective differences across mtDNA haplotypes have also been conducted using *D. melanogaster* and *D. simulans*. Kilpatrick and Rand (1995) tracked frequency changes of 2 mtDNA haplotypes from *D. melanogaster* (1 sourced from Argentina, the other Central Africa) when expressed alongside 3 distinct nuclear backgrounds (2 homozygous backgrounds that were associated to the 2 mtDNA haplotypes under study and 1 hybrid nuclear background) across replicate populations. Haplotype frequencies did not change on either of the 2 homozygous backgrounds, while the frequency of the Argentina haplotype increased only in the first 2 generations on the hybrid nuclear background but not in subsequent generations (Kilpatrick and Rand 1995).

Nigro (1994) set up 12 replicated population cages, testing the relative competitiveness of 2 mtDNA haplotypes from *D. simulans*, *si*II and *si*III, in 2 separate contexts—when *si*II had first been placed into the nuclear background of *si*III (via microinjection) or when it was introduced into the cages in its coevolved nuclear background. The results of these experiments were contingent on the nuclear context used. In cages where the haplotypes had been introduced via isofemale lines in which the original mitonuclear associations were preserved, the *si*II haplotype outcompeted the *si*III haplotype, but this pattern was reversed when the haplotypes were competing alongside the nuclear background originally associated with the *si*III (Nigro 1994), again suggestive of capacity for selection on mitonuclear genotype to mediate coadaptation between genomes.

Similarly, using haplotypes from *D. simulans* (*si*I, *si*II, and *si*III), Ballard and James (2004) tested for differences in the relative competitiveness of mtDNA haplotypes in population cage experiments. When population cages were seeded with equal starting frequencies of each haplotype, they found that the *si*II consistently increased in frequency across all replicate cages, whereas s*i*I decreased and s*i*III maintained its starting frequency. Following 15 generations of the experiment, the authors perturbed the haplotype frequency of each cage and then tracked ensuing haplotype frequencies across a further 28 generations of culture. They observed the same signature of positive selection on the *si*II haplotype, whereas *si*I was purged and *si*III maintained at lower population frequencies.

Rand (2011) sampled mtDNA haplotypes from a population of *D. melanogaster* in Texas, USA, which differed in their length only (short and long genome, in which the short haplotype was ∼1.4 kb shorter than the long form). Rand competed these haplotypes across 6 population cages—half initiated with high frequencies of the long variant, and the other half initiated with intermediate frequencies—but found no difference in the competitive abilities of the length variants across generations at the population level (Rand 2011).

In another study utilizing *D. melanogaster*, Wolff *et al*. (2017) established numerous experimental populations, each of which differed in their starting frequencies of 2 haplotypes—the male fertility-impairing Brownsville haplotype (discussed in the *Sex specificity of mitochondrial haplotype effects* section) and a control haplotype, Dahomey. Prior to the experiment, each of these haplotypes had been backcrossed independently into replicate mass-bred populations sourced from Dahomey; thus, the 2 haplotypes could be admixed at various starting frequencies and always competed against a heterogeneous nuclear background sourced from Dahomey. Rather than being set up in large cages, each population was maintained inside a 40-ml vial. This meant that population sizes were much smaller (*n* = 80 individuals per vial) than in a typical population cage (*n* = 100–1,000s per cage), but the benefit was that replication of experimental populations was high (84 populations per experiment). The Brownsville haplotype was seeded into populations at 4 different starting frequencies—0, 25, 50, and 75%, The authors observed that populations that were initially seeded with at least 50% of the Brownsville haplotype exhibited numerical suppression in population size over the course of 10 generations of monitoring, as a result of the negative effects of the Brownsville haplotype on male fertility. Notably, despite this population suppression effect, the frequency of the Brownsville haplotype increased across generations in these populations, exhibiting signatures of an adaptive response to positive selection (Wolff *et al.* 2017), presumably due to the fitness-augmenting effects that have previously been demonstrated to be associated with the same haplotype on adult females and juvenile flies (Wolff, Tompkins, *et al.* 2016; Camus and Dowling 2018). These effects were however only observed in populations maintained under density-controlled conditions and regulated population size and not when experiments were run under stochastic dynamics with fluctuating population sizes (Wolff *et al.* 2017).

Finally, Mossman, Ge, *et al.* (2019) competed 3 mtDNA haplotypes from *D. simulans/D. mauritiana* against each other, on 2 different *D. melanogaster* nuclear backgrounds, and in another set of experiments competed 2 haplotypes from *D. melanogaster* against each other, again on 2 *D. melanogaster* nuclear backgrounds, in a series of perturbation experiments across replicated population cages. They reported that haplotype frequencies in each set of experiments changed over time and that the changes differed according to the nuclear background. However, perturbation of allele frequencies at the midpoint of each experiment tended to change the trajectories of mtDNA haplotype changes, demonstrating generally poor repeatability in trajectories of mtDNA haplotype evolution and suggesting that there are no consistent differences in selection across time, on the alternative haplotypes within each species (Mossman, Ge, *et al.* 2019). Moreover, the rank order in performance of *D. simulans* haplotypes did not match those observed in the prior study by Ballard and James (2004), albeit an important difference between studies was that in Ballard and James (2004), the *D. simulans* haplotypes were competing on a native *D. simulans* nuclear background rather than that of *D. melanogaster* background used by Mossman, Ge, *et al.* (2019).

In sum, although the results of population cage studies to date have been mixed, several of these studies have identified signatures of selection on particular mtDNA haplotypes (Hutter and Rand 1995; Ballard and James 2004; Oliver *et al.* 2005; Arnqvist *et al.* 2016; Wolff *et al.* 2017), while results from other studies lack the consistency across replicate population cages required to disentangle effects of selection from effects of drift (MacRae and Anderson 1988).

## Insights from experimental evolution

The population cage studies reviewed above reported changes in population frequencies of mtDNA haplotypes in the absence of any dedicated treatments of laboratory natural selection. Yet, the emerging studies reviewed above have demonstrated that mitochondrial haplotype effects on performance are often sensitive to the intrinsic (e.g. sex or age) or abiotic (e.g. temperature or dietary variation) environment . These studies, reconciled by the results of population cage studies, have inspired researchers to test whether trajectories of mitochondrial haplotype evolution, within and across replicate populations, may respond adaptively to laboratory-imposed regimes of natural selection. This approach, known as experimental evolution, extends on classic population cage studies, typically through inclusion of a treatment of divergent natural selection (when testing for effects of directional selection) or a treatment of divergent starting frequencies of competing haplotypes (when testing for effects of negative frequency-dependent selection). Although the framework of experimental evolution provides one of the most powerful mechanisms to observe adaptive evolution in action and delineate adaptive from nonadaptive responses to natural selection (Kawecki *et al.* 2012), the technique has only rarely been applied to studying the capacity for evolutionary responses of mitochondrial variants to selection (Kazancıoğlu and Arnqvist 2014; Stojković *et al.* 2017; Aw *et al.* 2018; Lajbner *et al.* 2018; Kurbalija Novičić *et al.* 2020). Yet, several of the studies to apply the approach to this question have leveraged the *Drosophila* model.

Above (in *Intraspecific studies* section), we outlined a study of Camus *et al.* (2017), who had reported that 2 mtDNA haplotypes (A1 and B1) in *D. melanogaster* exhibit a latitudinal cline across the eastern seaboard of Australia, with fly strains harboring the A1 haplotype exhibiting heightened resilience to extreme heat stress but reduced tolerance to cold stress, relative to those with the B1 haplotype. These thermal tolerant attributes of the haplotypes aligned with their spatial distribution along the latitudinal cline; the A1 haplotype predominates in the subtropical north of Australia, B1 haplotypes in the temperate south. Based on these findings, it is predicted that that the A1 haplotype would have a selective advantage over B1 under warmer conditions and potentially respond adaptively by increasing in population frequency under such environments. To test this, Lajbner *et al.* (2018) leveraged the approach of experimental evolution, admixing flies harboring A and B group haplotypes into replicate populations, at intermediate frequencies. They then subjected replicate populations to 1 of 4 thermal selection treatments—constant warm (25°C) or cool (19°C) conditions or fluctuating warm (fluctuating with a mean of 26.4°C) or cool (fluctuating with a mean of 17.4°C) conditions. The experiments were conducted in duplicate, on a group of populations had either been treated with antibiotics (to purge infections with intracellular, cytoplasmically transmitted bacteria, principally *Wolbachia*, which is known to infect populations of flies in east coast Australia) and a group that remained untreated and that therefore carried *Wolbachia* infection. Consistent with previous findings of Camus *et al*. (2017), which had pointed to heightened tolerance of the A haplogroup to heat stress, Lajbner *et al*. (2018) observed that the A haplogroup increased in population frequency under the 2 warm (constant and fluctuating) treatments and decreased in frequency under the cooler treatments. However, these responses were only observed for population replicates that had been treated with antibiotics and were thus uninfected with *Wolbachia* (Lajbner *et al.* 2018). Similar to the A1and B1 haplotypes, frequencies of *Wolbachia* infection exhibit a latitudinal cline along east coast Australia, with infections at higher frequency at lower, subtropical latitudes (Kriesner *et al.* 2016), and there is accumulating evidence that these latitudinal patterns may be mediated by thermal selection, whereby the infection and spread of the bacterium within the *D. melanogaster* host is facilitated at warmer temperatures (Arnold *et al.* 2019; Truitt *et al.* 2019; Hague *et al.* 2022). It seems plausible that either interactions between temperature and *Wolbachia* (which, like the mtDNA, is similarly maternally inherited), or mild cytoplasmic incompatibility driven by varying levels of infection in the admixed populations, or complex interactions between mtDNA haplotype, *Wolbachia* and temperature, will have interfered with the capacity of the mtDNA to respond to selection in the presence of a *Wolbachia* infection.

Further work has identified putatively adaptive responses of mtDNA haplotypes to variation in dietary quality in *D. melanogaster*. Aw *et al*. (2018) tested the relative competitiveness of 4 haplotypes, expressed alongside an isogenic *w^1118^* nuclear background, to 4 diets differing in their ratio of protein to carbohydrates. Experiments were conducted in population cages over 12 generations. The authors observed shifts in the competitive performance of haplotypes across diets, with the “Alstonville” haplotype clearly increasing in frequency under a high ratio of protein to carbohydrate (1:2), but with this effect waning as ratios decreased. Indeed, at ratios of 1:16, Alstonville was outcompeted by a haplotype sourced from Dahomey. Further experiments conducted on these 2 haplotypes confirmed that switches in diets provided to population cages across the course of a 28 generation long experiment (from protein-to-carbohydrate ratios of 1:2 to 1:16 and then back to 1:2) resulted in corresponding changes in the population frequencies of the Alstonville and Dahomey haplotypes across population cages, with Alstonville always increasing under high protein and decreasing under low protein. The authors reported these frequency changes were mediated by differences between haplotypes in larval development across the 2 diets (Aw *et al.* 2018). This result is consistent with previous experiments on these same haplotypes that indicated both higher female reproductive success, and a higher larval viability, of Alstonville relative to Dahomey haplotypes, expressed alongside the same *w^1118^* nuclear background, when assayed on a similar high-protein diet (estimated at 1:3 protein:carbohydrate) (Camus and Dowling 2018).

Kurbalija Novičić *et al*. (2020) retested the question of whether negative frequency-dependent selection shaped the frequency of haplotypes I and II in *D. suboscura* (Oliver *et al.* 2005; Arnqvist *et al.* 2016), but this time starting the founding populations with different starting frequencies of each of the 2 haplotypes (commencing at either 20 or 80% of haplotype I) and then allocating the populations to each of 2 different food treatments—1 of homogenous food (3 identical dishes provided per cage containing standard cornmeal medium) and 1 of heterogenous food (3 dishes of cornmeal medium, each dish containing a different yeast concentration) treatment. Each combination of starting frequency and food treatment was replicated across 3 replicate populations. The authors observed that generally the rarer haplotype increased and the common haplotype decreased in frequency, across generations, converging on an equilibrium frequency, consistent with expectations of evolution under negative frequency-dependent selection. There was 1 exception however, with an apparent absence of response to selection in one of the treatment groups—the case in which populations were initiated with a starting frequency of haplotype I at 80% and then subjected to the heterogeneous food treatment (Kurbalija Novičić *et al.* 2020).

## Mitochondrial heteroplasmy—searching for an adaptive basis

Heteroplasmy describes the scenario in which 1 individual harbors multiple mtDNA haplotypes. Heteroplasmy can occur due to the accumulation of mtDNA mutations in the germline (a situation in which highly related mtDNA molecules that differ by 1 or a few polymorphisms will segregate within cells) or via biparental inheritance of mitochondria (a situation in which 1 individual could harbor 2 divergent mtDNA haplotypes). Heteroplasmy due to mutation accumulation is probably pervasive and can for instance explain the penetrance of many mitochondrial diseases in humans, in cases where mtDNA molecules carrying pathogenic mutations increase in frequency over time to the point in which they outnumber their wild-type counterparts within a given cell or tissue lineage, crossing a biochemical threshold at which disease is expressed (Stewart and Chinnery 2015). Heteroplasmy due to paternal leakage was in contrast, thought to be much rarer in nature, but there have been increasing reports of cases in metazoans, with theoretical and empirical work suggesting a possible adaptive basis to these cases (Van Leeuwen *et al.* 2008; Kuijper *et al.* 2015; Rollins *et al.* 2016; Allison *et al.* 2021).

Cases of heteroplasmy extend to *Drosophila*, through for example observation of biparental inheritance resulting from crosses between isofemale lines in *D. simulans*, in which a small proportion of mating pairs consistently produced heteroplasmic offspring (Wolff *et al.* 2013), and through observation of wild *D. melanogaster* sampled from European populations, where at least 14% of individuals were heteroplasmic, and the rate of paternal leakage (through sperm) estimated at 6% (Nunes *et al.* 2013). Heteroplasmy has also been observed to occur naturally in laboratory populations of *D. melanogaster*, with flies harboring mtDNA haplotypes that differ in the size of the control region (Kann *et al.* 1998). Experiments on these fly strains, differing in their control region length, suggested an age-specific transmission bias of the mtDNA haplotypes, with the longer haplotype increasing in frequency with incrementing female age. The longer haplotype was also enriched in mated females relative to virgin females, and furthermore flies that had laid their eggs at 25°C relative to those at 18°C had greater frequencies of the longer haplotype, suggestive of the hypothesis that selection may efficiently target mtDNA haplotypes competing under heteroplasmy (Kann *et al.* 1998).

Indeed, researchers have leveraged the experimental tractability of *Drosophila* to investigate the genetic forces that mediate changes in frequencies of haplotypes under heteroplasmy, within and across generations. Genetic strains carrying stable heteroplasmies can be created through microinjection of foreign germ plasm (from a donor with 1 mtDNA haplotype) into fertilized eggs (of a recipient that carries an alternative haplotype). de Stordeur *et al*. (1989) were among the first to establish this method in the fruit fly by generating reciprocal crosses of *D. simulans* that carried 2 divergent mitochondrial haplotypes (*si*II and *si*III) differing at about 1.5% of nucleotide sites. Assessing changes in heteroplasmy levels across 10 generations revealed a rapid decrease of *si*III haplotype frequency within the first 3 generations (de Stordeur *et al.* 1989). This directional shift, consistently favoring *si*II over *si*III, suggested that selection at the level of the cell could effectively target competing mtDNA haplotypes (de Stordeur *et al.* 1989). Concurrently, Matsuura *et al*. (1989) used microinjection to generate intraspecific and interspecific combinations of mitochondrial and nuclear genomes from *Drosophila* species, enabling their research group to then utilize these strains in a series of informative studies. Interspecific crosses, where *D. mauritiana* served as germ plasm donor and *D. melanogaster* as recipient, revealed a significant bias toward the fixation of the donor mtDNA when competing under heteroplasmy (Niki *et al.* 1989). Monitoring haplotype frequencies across 30 generations revealed a steady increase of donor mtDNA over the first 10 generations, after which the donor mtDNA went to fixation in 2 out of 4 strains and plateaued in the remaining 2 strains (Niki *et al.* 1989). Additional experiments in the following years included combinations of *D. melanogaster*, *D. simulans*, *D. mauritiana*, and *D. sechellia* serving as germ plasm donor and 4 recipient *D. melanogaster* strains (Matsuura 1991; Tsujimoto *et al.* 1991). In 1 study, the researchers maintained cohorts of each combination at 2 divergent temperatures (19°C and 25°C) to examine potential effects of thermal selection on the transmission and maintenance of mtDNA under heteroplasmy (Matsuura *et al.* 1990, 1991; Tsujimoto *et al.* 1991). These experiments confirmed nonrandom biases in mtDNA transmission, documenting selection favoring particular mtDNA haplotypes and showing that selective transmission of mtDNA differed depending on the temperature at which flies were maintained (Matsuura *et al.* 1991; Tsujimoto *et al.* 1991). Notably, transmission biases of mtDNA haplotype were contingent on both the nuclear background and temperature in both intraspecific and interspecific mitonuclear combinations and could result in selected biases toward the fixation of either donor or recipient mtDNA haplotypes (Niki *et al.* 1989; Matsuura 1991; Tsujimoto *et al.* 1991; Matsuura *et al.* 1993). A further study documented that shifts in heteroplasmy levels in strains involving pairwise combinations drawn from 3 lineages of *D. simulans* (carrying the *si*I, *si*II, or *si*III mtDNA type) and 2 strains of *D. mauritiana* (carrying the *ma*I or *ma*II mtDNA type) even exhibited a clear hierarchy in competitive haplotype advantage, with *si*II bearing the greatest advantage, followed by *si*III, *ma*I, *si*I, and finally *ma*II (de Stordeur 1997). De Stordeur (1997) also tested the effect of temperature on moderating frequency changes of 1 particular combination of *si*III/*ma*II haplotypes. In these experiments, changes in the temperature that the experiments were conducted at led to acceleration in the rate in which *si*III outcompeted *ma*II across generations, but not changes in the rank order of the heteroplasmy shifts.

While these microinjection experiments provided strong evidence for selection shaping frequencies of mtDNA haplotypes competing under heteroplasmy, the mechanisms underpinning the observed haplotype shifts remained unknown. It was proposed by Nagata and Matsuura (1991) that haplotype-specific optima of temperature-sensitive enzyme complexes that comprise the mETS and that are encoded in the mtDNA may lead to a selective advantage of 1 haplotype over another. However, analysis of several mETS components failed to detect significant differences in mitochondrial enzymatic activity in a subset of the heteroplasmic fly lines at the time, thereby suggesting that differences in temperature-dependent mitochondrial efficiencies were not the cause of the transmission biases of mtDNA in the examined strains (Nagata and Matsuura 1991). Instead, the biases seem to be under regulation of nuclear genes. Matsuura *et al*. (1997) created several heteroplasmic combinations of mtDNA haplotype, utilizing mtDNA haplotypes from *D. simulans*, *D. melanogaster*, and *D. mauritiana*, expressed in nuclear backgrounds from the respective 3 species. They then tracked changes in haplotype frequencies in the heteroplasmic strains, under 2 divergent temperatures, demonstrating consistent changes in haplotype trajectories across generations that were contingent on the nuclear background (Matsuura *et al.* 1997). Subsequent study by Doi *et al*. (1999) of a subset of the strains attempted to home in on the particular nuclear chromosomes involved in regulating the biases in mtDNA transmission across generations, by using chromosome substitution to interchange the second and third chromosomes of 2 different *D. melanogaster* strains and then examining changes in frequency of mtDNA of *D. mauritiana* when introduced in heteroplasmy into each newly constructed *D. melanogaster* strain. The results suggested that each of the chromosomes is involved in temperature-dependent selection in mtDNA transmission and by implication that genes across the 2 autosomal chromosomes (Doi *et al.* 1999) and probably the X chromosome as well (Matsuura *et al.* 1997) coordinate to regulate changes in mtDNA haplotype transmission under heteroplasmy.

Studies by Ma *et al.* (2014), Ma and O’Farrell (2016) and Chiang *et al.* (2019) further elucidated the mechanistic basis behind shifts in heteroplasmy levels in *Drosophila*. In a series of studies, the researchers worked with mutant mtDNA haplotypes, one of which contained a 9 base pair deletion in *mt:ND2* and another a temperature-sensitive mutation in the *mt:COI* gene in which flies are healthy at 25°C but die by 4 days of adult age at 29°C. They showed that strains heteroplasmic for both mutant mtDNA haplotypes were healthy at 29°C, thus revealing genetic complementation masking the mutant phenotypes, even when the temperature-sensitive haplotype was in very high abundance (∼95%). When the temperature-sensitive mtDNA haplotype was heteroplasmic alongside other wild-type haplotypes, high temperature selected for a decrease in the abundance of the mutant haplotype across generations, despite genetic complementation (Ma *et al.* 2014). Further work by Ma and O’Farrell (2016) indicated that outcomes of competition between heteroplasmic mtDNA pairs depended on levels of genetic divergence separating the haplotypes. Competition between closely related haplotypes (intraspecific combinations) favored haplotypes that conferred functional mETS, via efficient purifying selection. In contrast, competition between distantly related genomes (interspecific combinations) often favored haplotypes with negligible or even detrimental fitness consequences to the individual, indicating selfish selection via replication advantage even resulting in population death in 1 particular case (Ma and O’Farrell 2016). Finally, Chiang *et al*. (2019) conducted a genome-wide screen to home in on nuclear genes that influence the ratio of 2 mtDNA haplotypes, when competing in heteroplasmy: these were a functional haplotype from *D. yakuba* and a temperature-sensitive recombinant haplotype harboring the *mt:ND2* deletion and the temperature-sensitive *mt:COI* mutation from *D. melanogaster*. Heteroplasmy of this particular combination of haplotypes had been maintained stably over 70 generations, at a frequency of ∼5% *D. yakuba* mtDNA. The screen detected numerous nuclear modifiers of heteroplasmy levels, one of which was the catalytic subunit of the mtDNA polymerase gene (POLG), *tam*. The researchers showed that reducing expression of *tam* enhanced the effect of purifying selection via elimination of the mutant mtDNA recombinant haplotype. This effect of enhanced purifying selection upon downregulation of *tam* extended to other heteroplasmic combinations, resulting in consistent increases of the haplotype associated with higher mETS functionality (Chiang *et al.* 2019).

## Mitochondrial variation and mitonuclear interactions in health

It was in the 1980s that it was first discovered that mutations in mitochondrial genes can cause human disease (Wallace *et al.* 1988). In a landmark study, Wallace *et al.* (1988) found that a mutation in the mitochondrial tRNA^Lys^ compromised OXPHOS capacity, causing MERRF syndrome (myoclonic epilepsy with ragged red fibers), which can lead to expression of severe disease phenotypes, including hearing loss, exercise intolerance, and lactic acidosis. Since then, hundreds of mtDNA mutations have been implicated in a wide range of multisystem disorders, including aging (Larsson 2010), infertility (Ruiz-Pesini *et al.* 2000), or diabetes (Poulton *et al.* 2002). While in some instances, disease-causing mutations in mitochondrial genes have been identified (e.g. MERRF syndrome), often the search for the genetic cause of the mitochondrial-associated pathogenic mechanisms has been limited by observations of heterogeneity in disease penetrance and severity that are typically associated with mitochondrial disease.

Research utilizing *Drosophila* can help to uncover the mechanistic links between mitochondrial genotype and phenotype in human mitochondrial pathologies (for comprehensive reviews on the utility of *Drosophila* as human disease model, see Bier 2005; Bellen *et al.* 2010; Foriel *et al.* 2015; McGurk *et al.* 2015; Wangler *et al.* 2015; Sen and Cox 2017). High sequence similarity between *Drosophila* and human mitochondrial and nuclear genomes enables meaningful simulation and examination of human mitochondrial disease phenotypes in a tractable animal model organism with sufficiently complex organ system (Wangler *et al.* 2015; Sen and Cox 2017). Thus, many of the symptoms that accompany neurodegenerative and myodegenerative mitochondrial diseases can be recapitulated and examined in *Drosophila* models (Celotto *et al.* 2006, 2011; Burman *et al.* 2014).

For example, individuals affected by a missense mutation in the ATP6 gene or a 9-nucleotide deletion in the NADH dehydrogenase 2 gene exhibit symptoms resembling those typically observed in diseases such as Leigh syndrome and *M*itochondrial *E*ncephalomyopathy, *L*actic Acidosis, and *S*troke-like episodes (MELAS; Schon *et al.* 2001; Triepels *et al.* 2001; Celotto *et al.* 2011; Burman *et al.* 2014). Reflecting the typical progression of such diseases during the human life course, individual flies of the ATP6 model appear healthy after eclosion but reveal reduced locomotory activity at day 8, neuromuscular impairment at day 13, seizures at day 20, and premature mortality at day 30 (Celotto *et al.* 2011). Bioenergetic investigation of the ATP6 model at various stages of disease progression revealed that while the underpinning mutation in the ATP6 gene decreased ATPase activity in affected individuals, the bioenergetic shortfall was compensated for by increases in glycolysis, ketogenesis, and Kreb's cycle activity during early pathogenesis (Celotto *et al.* 2011). Analyses of the ND2 model also revealed reduced ATP levels but showed that the decrease in this model was caused by the reduced ability of OXPHOS complex I (in which ND2 resides) to efficiently couple electron transfer to proton pumping, thereby diminishing mitochondrial membrane potential and thus ATPase activity (Burman *et al.* 2014). In another *D. melanogaster* model, a similar disruption of electron flux caused by a temperature-sensitive nonsynonymous mutation in the cytochrome c oxidase 1 (mt:CO1) gene could be rescued by expression by a GAL4/UAS-enabled alternative oxidase (Hill *et al.* 2014; Chen *et al.* 2015). And in another example, effects resembling mitochondrial disease (decrease activities of OXPHOS complexes I–IV, developmental delays, stress-induced seizures, and defective male courtship behavior) associated with a mutation (tko^25t^) in the nuclear gene-encoding mitoribosomal protein S12 of *D. melanogaster* were shown to be sensitive to the mtDNA haplotype background alongside which the tko^25t^ was expressed. Of 4 mtDNA backgrounds studied, 1 particular mtDNA background accentuated the negative effects of tko^25t^, leading to high numbers of melanotic modules and hemolymph abnormalities, indicators of defective innate immunity, and developmental lethality (Salminen *et al.* 2019).

Together, these experiments have provided insights into the dynamic metabolic changes and compensatory mechanisms accompanying disease progression and highlighted the utility of fly models to conduct time course studies throughout the lifetime of affected individuals to help elucidate disease pathways relevant to humans. Even in cases in which disease phenotypes are difficult to simulate, such experiments show how aspects of mitochondrial dysfunction that form part of specific human pathologies can be recapitulated in a fly model, and that systematic analysis of pathological outcomes starting from the organismal phenotype via cytological changes through to molecular mechanisms can enable dissection of complex disease traits.

Despite proven potential, the number of *Drosophila* models that specifically investigate mutations in the mitochondrial genome in the expression of disease is still limited (Sen and Cox 2017). This is somewhat surprising considering that the literature is rich with studies that have documented broad effects of mitochondrial haplotypic variation on the expression of fundamental fitness traits, many of which could potentially serve as disease models (e.g. fertility, physical activity, longevity, and stress resistance; see *Mitochondrial variation, mitonuclear interactions, and life history—interpopulation (intraspecific) and interspecific tests* section). One challenge to further development of *Drosophila* as a model for the study of mitochondrial disease will hinge on the ability to unambiguously identify and isolate single disease-causing mutations in the mtDNA sequence. The most effective avenue to achieve this will be through development of mitochondrial genome editing to disassociate single mutations within haplotypes linked to disease.

While genome-editing tools have already been used to establish the ATP6 and ND2 models discussed above (Burman *et al.* 2014; Chen *et al.* 2015), targeted modification of mtDNA has remained largely restricted to specific sequence motifs, thereby limiting the scope of this approach (Xu *et al.* 2008). While the CRISPR-Cas9 gene-editing system has proven revolutionary in enabling researchers to edit any nucleotide within a gene within the nuclear genome (Doudna and Charpentier 2014) and examine the associated effects on function, this system has not been compatible to the mtDNA sequence because RNA guides have not been able to be developed that enter into the mitochondrion (Falkenberg and Hirano 2020). Notwithstanding, several developments have emerged in recent years that suggest that mitochondrial gene editing is now eminently within reach for the future study of mitochondrial biology in the *Drosophila* model (Silva-Pinheiro and Minczuk 2022). This includes the development of DddA-derived cytosine base editors (DdCBE), derived from an interbacterial toxin, which are able to catalyze conversions of C to G and T to A nucleotides within the mtDNA with high specificity in mammals (Mok *et al.* 2020; Silva-Pinheiro *et al.* 2022).

These gene-editing approaches will likely prove game changing to our capacity to understand links between mtDNA variants and health. Development and application of these technologies to the experimentally tractable *Drosophila* model will provide an invaluable tool for homing in on the modulators of mitochondrial disease penetrance. These technologies will enable effects of specific mtDNA mutations to be assessed across an array of different nuclear and mitochondrial genotypic backgrounds and environmental contexts, homing in on the capacity for genetic compensatory mechanisms to evolve that offset the effects of the mutations and elucidating the interplay between mtDNA variation and environment in shaping health.

Similarly, currently available gene-editing technologies could provide a powerful method to investigate the effects of nuclear-encoded mutations that confer mitochondrial disease when expressed against different mtDNA haplotypes, to determine the generality by which mtDNA haplotype backgrounds moderate disease penetrance, as well as when expressed against diverse nuclear backgrounds and environmental contexts. Such studies are likely to reveal new insights into the capacity for mitonuclear interactions to moderate the penetrance of known disease-causing mtDNA pathogenic mutations.

## Conclusion and outlook

Research over the past 3 decades has changed the way in which we view the mitochondrial genome and the genetic variation therein (Ballard and Whitlock 2004; Rand *et al.* 2004; Dowling *et al.* 2008; Wallace 2010; Lane 2011; Pesole *et al.* 2012; Horan *et al.* 2013; Wolff *et al.* 2014; Sloan *et al.* 2017; Hill *et al.* 2019). Today, the importance of mitochondrial genetic variation in moderating the expression of organismal fitness is broadly recognized, and evidence continues to accumulate to suggest that this variation may contribute to outcomes of ecological and evolutionary processes (Blier *et al.* 2001; Mishmar *et al.* 2003; Ballard and Whitlock 2004; Dowling *et al.* 2008; Gershoni *et al.* 2009; Burton *et al.* 2013; Wolff *et al.* 2014) and the expression of complex human diseases (Wallace 2010, 2012; Horan and Cooper 2014; Picard *et al.* 2016). Many of the insights that have contributed to advancing this field of research were facilitated through work in *Drosophila*. Despite the small size and a generally perceived deep knowledge of the mitochondrial genome, its gene content is currently undergoing scrutiny. Deep-sequencing approaches and gene expression experiments have uncovered several protein-coding genes implicated in modifying the expression of complex human disease (e.g. Alzheimer's and diabetes; Tajima *et al.* 2002; Lee *et al.* 2013; Lee *et al.* 2015), sex-specific transmission of mitochondrial genomes in some bivalves (Breton *et al.* 2011), and mitochondrial peptides and RNAs with yet unknown function (Faure *et al.* 2011; Mercer *et al.* 2011; Seligmann 2012, 2013; Pozzi and Dowling 2019, 2021, 2022). The extent to which similar genetic elements exist in the mitochondrial genome of *Drosophila* species remains to be reported, but the increase in frequency at which such elements are discovered across taxa suggests that the genetic repertoire and complexity of the mitochondrial genome may have previously been underestimated. Notwithstanding, the function of these genetic elements remains largely elusive and demanding of experimental approaches that test their functionality—research questions that are perfectly suited to study through the *Drosophila* model.

Much remains to be discovered when it comes to understanding the evolutionary implications of mitochondrial genetic variation, across all levels at which selection can act to shape patterns of variation—from heteroplasmy within cells and tissues of individuals, to populations and species. Currently, it is clear that the link between mitochondrial genotype and phenotype is routinely moderated by the nuclear genetic background, via mitonuclear interactions, the outcomes of which can be mediated by heterogeneity in the intrinsic (e.g. sex and age) and extrinsic environments (e.g. climate and diet). Whether or not these epistatic interactions can mediate evolutionary responses to natural selection within populations or patterns of reproductive isolation between incipient species remains open to question and would benefit from attention of further studies that embrace approaches such as experimental evolution, which enable evolutionary trajectories to be tracked in real time. The evolutionary and ecological implications of sex-specific mitochondrial genetic variation also require further attention: firstly, to clarify the generality by which such sex specificity is male biased and consistent with predictions of the Mother's Curse hypothesis; and secondly, to determine whether such sex specificity may contribute to dynamics of sexually antagonistic coevolution or outcomes of sexual selection generally.

Moreover, the underpinning loci involved in mitonuclear interactions remain largely elusive, and identifying these loci should be a priority for future research. Researchers are increasingly embracing next-generation sequencing technologies to dissect the functional basis of mitonuclear interactions at the genome-wide scale, revealing new insights into the broader influence of mitochondrial sequence variation in the regulation of the nuclear gene expression and the mechanisms linking mitochondrial genotype to phenotype (Torres *et al.* 2009; Innocenti *et al.* 2011; Rogell *et al.* 2014; Mossman, Tross, *et al.* 2016; Mossman, Biancani, *et al.* 2019; Ågren *et al.* 2020). Further development of genome-editing technologies that enable direct modification to mtDNA sequences will ultimately overcome the problem that researchers currently face in partitioning independent effects of different genetic polymorphisms within a genome in which all loci are in complete linkage; such technologies will ultimately enable precise dissection of individual mtDNA polymorphisms to unambiguously identify functional variants involved in complex genomic and environmental interactions mediated by the mitochondrial genome. The *Drosophila* model is ideally positioned to address the outstanding questions of the future and further advance understanding of the ecological and evolutionary significance of mitochondrial genetic variation.
